# IoT-based smart water billing system for accurate consumption monitoring and fair distribution

**DOI:** 10.1038/s41598-026-58773-8

**Published:** 2026-07-18

**Authors:** M. Mokhtar Zayed, Mohamed A. El-morsy, M. H. Shaker, Mohamed Edries

**Affiliations:** 1 Department of Communications, Faculty of Electronic Engineering, Menoufia University, Menouf City, Menoufia Governorate Egypt; 2 Department of Communications and Computers Engineering, Higher Institute of Engineering, El-Shorouk Academy, El-Shorouk City, 11837 Cairo Governorate Egypt

**Keywords:** Smart water meter, IoT, Automated billing, Flow measurement, Leak detection, Water conservation, Real-time monitoring., Energy science and technology, Engineering, Mathematics and computing

## Abstract

Accurate water consumption measurement and equitable billing are vital for sustainable water resource management, especially in regions facing scarcity and uneven distribution. Conventional systems, which often rely on shared mechanical meters, fail to provide individual usage data, leading to disputes, waste, and inefficiencies. This paper presents the design and experimental validation of a low-cost, IoT-enabled Smart Water Billing System (SWBS) that delivers real-time consumption monitoring, adaptive calibration, and automated proportional billing. The proposed system integrates an ESP8266 NodeMCU, YF-B1 Hall-effect flow sensors, and anomaly detection algorithms to ensure precise measurement and early leak detection, where the IoT functionality of the prototype is realized through the Wi-Fi-enabled NodeMCU, which transmits processed consumption and status data to a remote backend using HTTP-based communication for cloud-connected logging and monitoring. A multi-stage calibration method was developed to maintain accuracy across diverse flow rates, reducing measurement error to below 3%. Experimental results confirm that the system reliably tracks individual unit consumption, supports scalable deployment in multi-unit buildings, and provides transparent billing through wireless data transmission. By combining affordability, accuracy, and real-time analytics, the SWBS offers a sustainable solution for modern urban water management, promoting conservation and fair resource distribution.

## Introduction

Water is one of the most essential yet finite natural resources, and its sustainable management has become a global priority due to the growing challenges posed by rapid population growth, urbanization, and climate change impacts^1,2^. Urban water distribution systems face increasing pressure to provide equitable access while minimizing wastage and operational losses. Accurate measurement of water consumption is a fundamental requirement for effective management, as it directly enables fair billing, early leak detection, and the development of informed conservation strategies^3,4^. In recent years, smart metering technologies have transformed the way utilities monitor and bill water usage by integrating real-time data acquisition, wireless communication, and advanced analytics, thereby enhancing both operational efficiency and consumer engagement^5,6^. However, in many developing countries, including Egypt, water billing systems still rely on traditional mechanical meters, with a single meter often shared across multiple units within a building. Under this model, the total water bill is split equally among residents, regardless of their actual individual consumption^7,8^. This practice not only fails to represent real usage patterns but also discourages conservation efforts and often leads to conflicts among tenants. Furthermore, mechanical meters are prone to degradation over time, sediment accumulation, and limited resolution, while manual readings introduce human errors and delays^9,10^. IoT-based smart water meters have emerged as a promising solution to these challenges by enabling real-time monitoring, automated meter reading, and two-way communication between consumers and utilities^11–13^. These systems allow users to track water usage at a granular level, detect leaks promptly, and optimize distribution networks to reduce losses. Several research efforts have explored IoT-based water monitoring and billing solutions, leveraging wireless sensor networks, cloud computing, and mobile applications to provide continuous consumption tracking and anomaly detection^14–18^. Despite these advancements, many existing implementations face limitations such as a lack of adaptive error correction, which is critical for maintaining accuracy under real-world conditions where water flow rates vary significantly. Without dynamic calibration, sensor readings may deviate considerably at low or high flow rates, compromising both billing precision and leak detection capabilities.

To address these limitations, this paper proposes the design and development of a low-cost, IoT-enabled Smart Water Billing System (SWBS) that enables real-time individual unit monitoring, adaptive multi-stage calibration, leak detection, and equitable proportional billing. The system integrates an ESP8266 NodeMCU Wi-Fi module, inline YF-B1 Hall-effect flow sensors for branch-line water measurement, auxiliary infrared (IR) proximity sensing for contextual activity indication, and centralized data processing. Each sensing unit is intended to be installed on the household branch water line feeding the corresponding user outlet, enabling per-user consumption monitoring without requiring direct mounting on the faucet body. In the implemented prototype, the ESP8266 performs local sensing, calibration, and anomaly checking, and then transmits the processed measurements to a remote backend through Wi-Fi communication using HTTP POST over the TCP/IP protocol stack, thereby enabling IoT-based remote monitoring and data logging. By applying a multi-stage calibration process, the SWBS achieves consistent accuracy across different flow ranges, while anomaly detection algorithms identify potential leaks and unauthorized water usage. This integrated approach ensures both technical performance and affordability, making it suitable for large-scale deployment in residential and urban water distribution networks.

### Motivation and key contributions

The motivation for this research stems from the urgent need to improve water billing fairness, reduce wastage, and empower consumers with actionable insights. In urban contexts with shared meters, fair billing based on actual usage can foster conservation behavior and reduce unnecessary consumption. Integrating IoT capabilities enables utilities to operate more efficiently while offering end-users transparency and control over their consumption. 

The main contributions of this work can be summarized as follows. First, an IoT-based smart water billing system is designed and implemented by integrating flow sensing, wireless communication, and real-time monitoring within a unified low-cost platform. Second, an adaptive calibration strategy is developed to improve measurement accuracy across varying flow conditions. Third, the system incorporates anomaly-detection and leakage-identification logic to support more reliable water-resource management. Fourth, experimental validation demonstrates that the proposed platform achieves a measurement error below 3% after calibration. Finally, the overall architecture is designed to support multi-unit residential deployment and to provide a foundation for future extension toward larger smart-water infrastructures. 

The remainder of this paper is organized as follows: Sect. 2 reviews relevant literature on IoT-based smart water monitoring and billing systems. Section 3 describes the proposed SWBS architecture, including its hardware, software, and mathematical models. Section 4 discusses the implementation and testing methodology, while Sect. 5 presents the experimental results and analysis. Finally, Sect. 6 concludes the paper and outlines future research directions.

## Literature review

The growing demand for safe, efficient, and sustainable water resource management has driven extensive research into IoT-enabled monitoring, billing, and optimization systems. One major research focus has been water quality monitoring combined with smart billing mechanisms. Nivedan and Poornima^19^ developed a system integrating pH, turbidity, and conductivity sensors to ensure the distribution of potable water only, with a solenoid valve controlling flow and an IoT platform enabling continuous quality assessment. Their system billed users only for water meeting safety standards, thereby reducing waterborne disease risks and promoting hygiene. Similarly, Lakshmikantha et al.^24^ proposed an IoT-based real-time water quality monitoring system capable of continuous measurement and cloud-based data analysis, issuing early alerts in cases of contamination. Complementing these implementation-oriented studies, Zulkifli et al.^25^ presented a large-scale review of water quality monitoring research published between 2018 and 2022, identifying critical gaps in data acquisition systems and emphasizing the need for intelligent and generalized frameworks capable of delivering accurate, real-time quality assessments across diverse locations and environmental conditions.

Another important research direction addresses household-level consumption monitoring and behavior change. Yang et al.^20^ advanced appliance-level monitoring through the FP7 ISS-EWATUS project by combining smart metering with a Decision Support System (DSS) to provide real-time consumption insights and tailored water-saving recommendations. Their field implementation in Poland and Greece demonstrated measurable changes in user behavior toward conservation. Likewise, Rosyady et al.^34^ developed an IoT-based water consumption management system with monitoring and control capabilities, enabling users to track daily usage, prevent overflow through automated pump control, and maintain a consumption history. Together, these studies highlighted the importance of consumer engagement and feedback loops in reducing water wastage.

Energy self-sufficiency in smart metering systems has also been extensively studied through energy harvesting technologies. Hoffmann et al.^21^ demonstrated that a rotational radial-flux harvester embedded in a conventional meter housing could generate up to 720 mW from water flow, while Kong^22^ explored Vortex-Induced Vibration (VIV) as a means of converting hydrokinetic energy into electricity, reporting 41 mW under high-flow conditions. Similarly, Boisseau et al.^26^ designed a horizontal-axis turbine with distributed magnets, producing up to 25.2 mW while minimizing pressure loss. Li and Chong^27^ integrated a turbine generator into a self-powered smart water meter that combined flow sensing and battery charging, whereas Pimenta and Chaves^29^ retrofitted mechanical meters with Narrowband Internet of Things (NB-IoT)communication and hybrid energy harvesting to reduce reliance on battery replacement. Collectively, these studies demonstrate the feasibility of sustainable energy harvesting for smart metering, although efficiency under low-flow conditions remains a common challenge.

A significant body of research also explores IoT architectures and smart water management frameworks for smart cities. Shahanas and Sivakumar^23^ proposed an IoT-based campus-scale water monitoring system with a future pathway toward city-wide deployment. Palermo et al.^28^ integrated smart water management into building systems to optimize consumption and promote reuse strategies such as rainwater harvesting. Syrmos et al.^35^ presented a modular IoT architecture capable of both quantitative and qualitative monitoring, while supporting scalability and low-cost deployment in both urban and rural environments. Soares Ascenção et al.^36^ reviewed 100 smart water management applications and highlighted the strong interconnections among smart metering, loss management, and consumption monitoring. In addition, Okoli and Kabaso^38^ reviewed IoT communication technologies for smart water applications and emphasized the potential of Low-Power Wide-Area Network (LPWAN) solutions for long-range and low-power deployment.

Finally, advances in automated metering and intelligent data processing have significantly improved operational efficiency. Omer^30^ proposed a Global System for Mobile Communications (GSM-based Automatic Meter Reading (AMR) system for real-time billing and remote service management, while Lee et al.^31^ introduced a Bidirectional Long Short-Term Memory (BiLSTM) autoencoder for anomaly detection in multi-utility metering data and reported improved performance over conventional Long Short-Term Memory (LSTM)-based methods. Amaxilatis et al.^32^ further showed that advanced data-imputation techniques, including deep learning models, can improve the reliability of water-consumption datasets, thereby supporting leak detection and predictive maintenance. In the broader context, Gupta et al.^33^ reviewed the role of Smart Water Technology (SWT) in reducing losses and optimizing water usage across sectors, whereas Zapata-Sierra et al.^37^ provided a bibliometric analysis of smart water metering research and identified emerging themes such as machine-learning integration for real-time network management.

Recent contributions further strengthen the role of intelligent analytics and field-ready deployments in anomaly and leak detection. Kanyama et al.^39^ showed that end-to-end machine-learning pipelines with data resampling can handle class imbalance effectively, thereby improving anomaly recall and operational stability in real deployments. Rezaiezadeh Roukerd and Rajabi^40^ highlighted the growing role of supervised and unsupervised machine-learning models in smart water systems, particularly for forecasting and anomaly flagging. Santos-Fernandez et al.^41^ proposed unsupervised spatio-temporal methods that improve robustness across heterogeneous meter types and districts while reducing dependence on labeled datasets. In large-area deployment contexts, recent LoRaWAN-based Advanced Metering Infrastructure (AMI) demonstrations^42^ confirmed the feasibility of wide-area metering with faster leak localization and lower operational overhead. Jamadarkhani et al.^43^ further showed that non-intrusive and multi-sensor fusion approaches can improve detection robustness in intermittent water networks. More broadly, recent reviews by Taloma et al.^44^ and studies on hybrid leak-localization models^45^ indicate a strong transition toward unsupervised or semi-supervised detection combined with edge intelligence and multivariate sensing.

Collectively, these studies establish the state of the art in IoT-based smart water monitoring, energy harvesting for self-sufficiency, data analytics for operational optimization, and consumer engagement for conservation. However, most existing systems either focus on a single aspect such as quality monitoring, consumption tracking, or energy harvesting or lack adaptive error correction across varying flow rates. The current study addresses these gaps by developing a low-cost, IoT-enabled SWBS capable of precise per-unit monitoring, adaptive calibration, anomaly detection, and proportional billing, thereby integrating multiple advanced features into a unified, deployable solution.

The reviewed literature is organized into two complementary tables to clearly distinguish between different types of contributions. Table 1 presents implementation-oriented studies in smart water monitoring, billing, anomaly detection, and energy-aware metering, focusing on their methods, results, and technical limitations, while Table 2 summarizes review, survey, and bibliometric studies, highlighting their scope, synthesis contributions, and identified research gaps. This separation was adopted because combining experimental implementations and knowledge-synthesis works in a single table could obscure key differences in their nature and objectives, whereas the two-group structure provides a clearer and more meaningful comparison.

**Table 1:** Comparison of implementation-oriented studies related to smart water monitoring, metering, and billing.

Ref.	Application/System Type	Key Methods/Technologies	Main Results/Contributions	Main Limitation
^19^	Potable-water supply with smart billing	pH, turbidity, and conductivity sensing; solenoid valve; IoT monitoring	Enabled billing only for water classified as potable; improved water-quality-aware delivery	Limited scalability testing
^20^	Appliance-level household water monitoring	Wireless appliance-level meters; Decision Support System (DSS); user feedback loop	Demonstrated measurable water-conservation behavior in field deployment in Poland and Greece	Strong dependence on user engagement
^21^	Energy harvesting for underground smart meters	Rotational radial-flux generator integrated with meter housing	Generated up to 720 mW at 20 L/min, showing the feasibility of self-powered metering support	Poor efficiency below 5 L/min
^22^	Hydrokinetic energy harvesting for water systems	Bluff body with piezoelectric harvester using Vortex-Induced Vibration (VIV)	Produced up to 41 mW under high-flow conditions	High dependence on turbulence and flow conditions
^23^	Campus-scale smart water monitoring	IoT-based water tank monitoring architecture	Feasible Demonstrated practical feasibility for small-scale deployment with potential expansion pathway-scale deployment	Focus limited mainly to tank monitoring
^24^	Real-time water quality monitoring	IoT sensors; cloud analytics; remote monitoring	Enabled continuous quality assessment and early contamination alerts	Limited monitored parameter diversity
^26^	Low-loss turbine energy harvester for metering	Horizontal-axis turbine with distributed magnets	Achieved minimum pressure loss and about 25.2 mW output	Limited field validation
^27^	Self-powered smart water meter	Turbine generator; Bluetooth-enabled monitoring	Supported operation over 200–650 L/h with mobile monitoring capability	Short communication range
^28^	Building-level smart water optimization	IoT devices integrated with building management systems (BMS)	Reduced losses and promoted reuse strategies such as rainwater harvesting	Building-scale focus only
^29^	Retrofitting conventional mechanical meters	NB-IoT communication; hybrid energy harvesting	Achieved stable and accurate meter retrofitting with reduced battery dependence	Indoor light harvesting remained weak
^30^	Remote water billing and service control	Arduino-based Global System for Mobile Communications (GSM) Automatic Meter Reading (AMR)	Enabled remote billing and control functions	Dependent on GSM network availability
^31^	Anomaly detection in utility metering	Bidirectional Long Short-Term Memory (BiLSTM) autoencoder	Outperformed conventional LSTM-based anomaly detection	Requires labeled training data
^32^	Data recovery/reliability enhancement in smart meters	Artificial-intelligence-based data imputation	Achieved low mean absolute error, reported as low as 0.16	Performance depends on method-specific tuning
^34^	IoT-based home water monitoring and control	Arduino platform; tank-level monitoring; pump control	Prevented overflow and supported usage history; reported about 6.16% error	Accuracy degraded with increasing volume
^35^	Modular IoT water monitoring system	LPWAN-compatible modular architecture; quantitative and qualitative sensing	Supported scalable, low-cost monitoring architecture for urban and rural contexts	Only initial pilot validation
^39^	AI-driven anomaly detection in smart water metering networks	End-to-end machine-learning pipelines; data resampling for class imbalance	Improved anomaly recall and leak-detection stability in real deployments	Requires continuous retraining/updating
^41^	Unsupervised anomaly detection in water metering	Spatio-temporal clustering of flow and pressure signals	Improved robustness to sparse labels and concept drift	High computational cost at scale
^42^	Large-scale Advanced Metering Infrastructure (AMI)	LoRaWAN-based IoT meters; edge analytics; cloud integration	Enabled efficient wide-area data collection and early leak detection	Limited testing under dense urban interference conditions
^43^	Multi-sensor smart metering for intermittent networks	Fusion of pressure, flow, and valve-state sensing	Improved anomaly/leak detection accuracy in intermittent supply conditions	Higher installation and integration complexity
^45^	Leak detection and localization	Hybrid machine-learning methods using pressure and flow data	Achieved higher F1-score for leak localization in bench/field settings	Higher deployment cost in large networks

**Table 2:** Comparison of review, survey, and bibliometric studies related to smart water systems.

Ref.	Study Type	Scope/Focus	Key Insights	Relevance/Limitations
^25^	Systematic review	IoT-based water monitoring systems (2018–2022)	Identified gaps in data acquisition systems and real-time monitoring frameworks	Strong background, but no implementation
^33^	Literature review	Smart Water Technology (SWT)	Highlighted benefits and challenges of smart water technologies in resource management	Broad overview only
^36^	Literature review	Smart water management applications	Identified 100 applications across metering, loss control, and monitoring	No unified implementation framework
^37^	Bibliometric review	Scientific trends in smart water metering	Identified emerging themes such as ML integration and network intelligence	Does not compare technical performance
^38^	Systematic review	IoT communication technologies for smart water systems	Highlighted LPWAN potential for long-range and low-power deployment	Limited emphasis on implementation benchmarking
^40^	Review of ML methods	ML for smart water systems	Summarized forecasting and anomaly-detection approaches	Dependent on existing reported studies
^44^	Review of ML in smart water systems	Edge ML, unsupervised methods, anomaly analytics	Highlighted shift toward edge intelligence and unlabeled anomaly detection	Limited real-world deployment evidence

As shown in Tables 1 and 2, prior implementation-oriented studies have often focused on a limited subset of functionalities, such as quality monitoring, household-level usage tracking, anomaly detection, or energy harvesting, whereas review- and survey-oriented studies have highlighted broader challenges related to scalability, communication architecture, intelligent analytics, and deployment readiness. This comparison further supports the need for an integrated, low-cost smart water billing platform that combines adaptive calibration, anomaly detection, and building-level billing reconciliation within a single deployable framework.

Across prior studies^19–45^, several limitations persist:

1. Flow-range bias & sensor nonlinearity: Many systems use a single global factor for Hall-effect meters, leading to larger errors at low/high flow extremes; few adopt piecewise or adaptive calibration robust across 1–30 L/min. Our work directly addresses this through multi-stage calibration and verified < 3% error across ranges.
2. Label scarcity & drift: Real deployments rarely have labeled anomalies; models often degrade under concept drift (seasonality, usage shifts). While unsupervised methods are rising, deployment-ready pipelines with on-device (edge) calibration and drift adaptation are still uncommon.
3. Building-level reconciliation: Few implementations validate per-flat totals against a main meter in real time and enforce billing consistency checks under realistic multi-user scenarios. Our multi-meter consistency formulation and validation fill this gap.
4. Actionable leak rules + robust Machine Learning (ML): Pure ML often lacks operational safeguards (e.g., presence sensors, rule-based lockouts). Our combination of Cumulative Sum (CUSUM) + rule-based IR presence checks with controllable H-bridge cut-off provides a safety-critical layer beyond analytics.

## System architecture

**Fig. 1:**
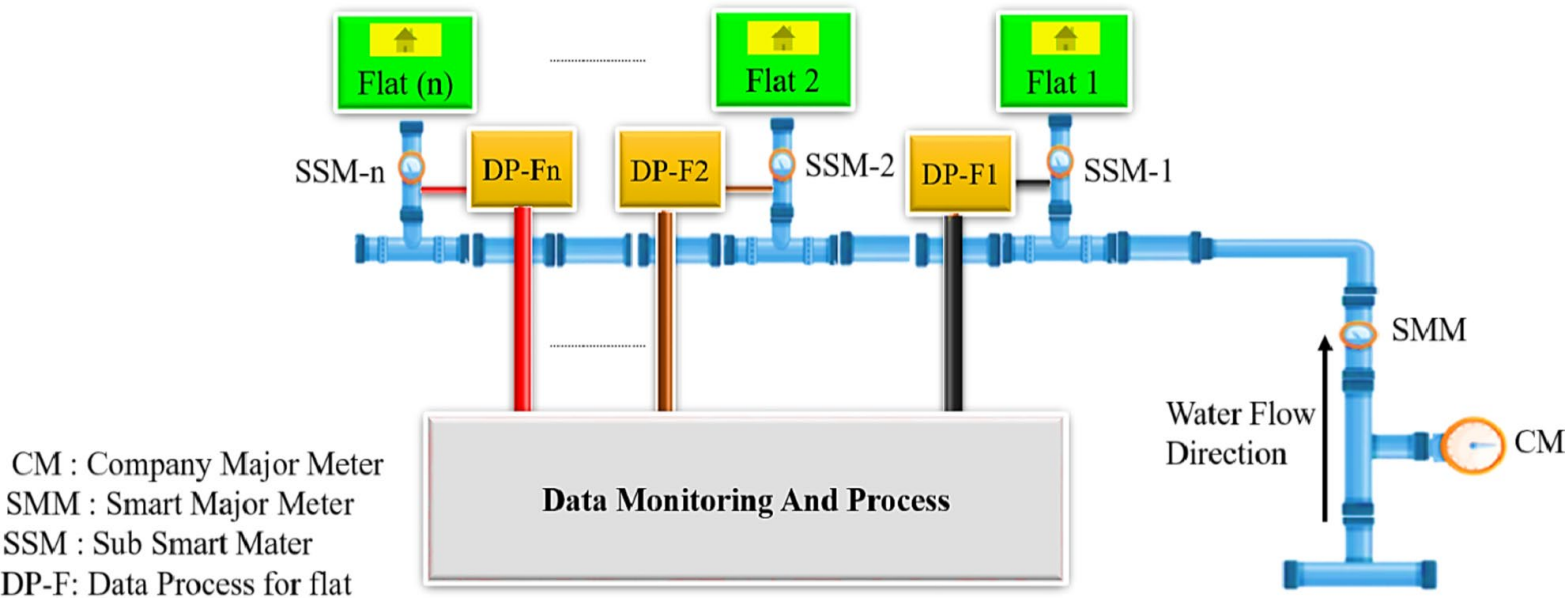
The system architecture of the proposed SWBS.

The proposed Smart Water Billing System (SWBS) is designed to provide real-time, accurate monitoring of water consumption for multi-unit residential buildings while supporting scalability for city-wide deployments. Its architecture integrates low-cost sensing modules, wireless communication, centralized data processing, and automated anomaly detection, ensuring precise measurements and equitable billing. Figure 1 presents the system architecture of the proposed SWBS designed for multi-unit residential buildings. Each flat (Flat 1, Flat 2, … Flat n) is connected to the main water distribution line through a dedicated inlet equipped with a flow sensor and a digital processing unit (DP-F1, DP-F2, … DP-Fn). These units are responsible for recording pulse signals generated by the flow sensors, converting them into volumetric consumption, and transmitting the data to a central monitoring and processing module. The central unit aggregates the measurements, validates their consistency against the main supply meter, and applies calibration and anomaly detection algorithms to ensure accuracy and reliability. This architecture guarantees that each household’s water consumption is independently monitored, thereby enabling equitable billing based on actual usage rather than a shared estimate. Furthermore, the scalability illustrated in the figure allows the system to support any number of units within a building, while the central data processing module facilitates both proportional and tiered billing schemes. In this way, Fig. 1 encapsulates the integration of sensing, processing, and billing that underpins the novelty and fairness of the proposed system.

Key architectural benefits include:

- **Scalability**: Easily expandable from single buildings to entire districts by adding new nodes.
- **Accuracy**: Adaptive calibration minimizes errors across a wide flow range (1–30 L/min).
- **Resilience**: Local processing ensures functionality even during temporary internet outages.
- **Actionable control**: Integration with pumps and relays enables automated cutoff in case of anomalies.

### System block diagram

**Fig. 2:**
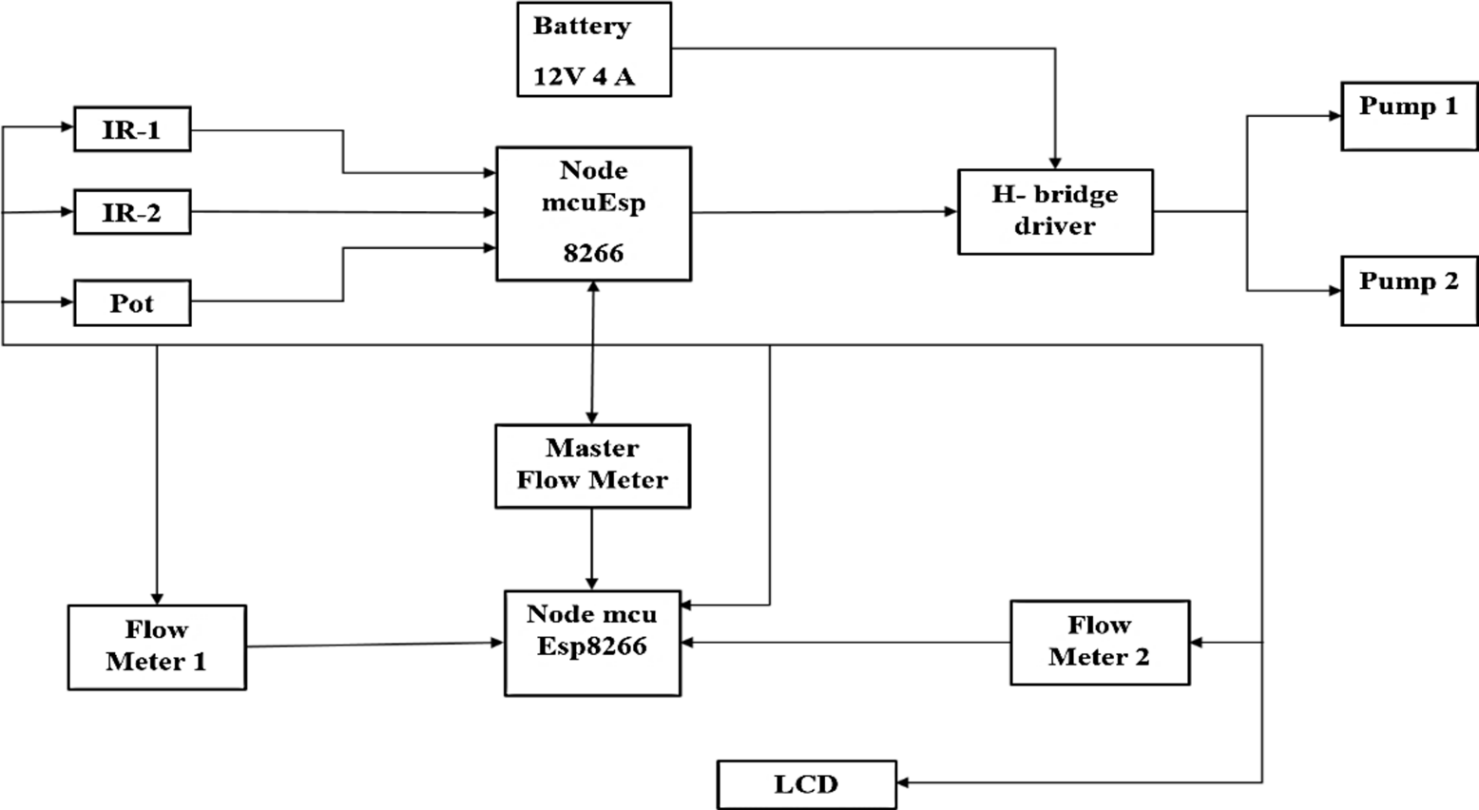
The hardware block diagram of the proposed SWBS.

Figure 2 illustrates the hardware block diagram of the proposed SWBS, which integrates sensing, control, and communication modules into a unified IoT-enabled platform. From an IoT perspective, the ESP8266 NodeMCU functions as a wireless edge device that acquires local measurements, executes embedded processing, and relays the processed data through the network to a remote monitoring backend. At the core of the system is the NodeMCU ESP8266 microcontroller, which serves as the central hub for local sensing, embedded data processing, and wireless communication. The ESP8266 collects pulse signals from the flow sensors, converts them into flow and consumption values, applies the local calibration and anomaly-detection logic, and then transmits the processed data through its built-in Wi-Fi interface to a remote server. The communication is performed using HTTP POST requests over the TCP/IP protocol stack, which enables cloud-connected monitoring and supports the IoT functionality of the proposed platform. From a data-flow perspective, the operation begins with pulse generation at the YF-B1 flow sensor, which measures the passing water volume in the branch line. These pulse signals are read by the ESP8266 NodeMCU, which performs pulse counting, flow-rate estimation, adaptive correction, and anomaly evaluation in real time. The resulting consumption values and alert states are then routed to the LCD display for local visualization and, simultaneously, to the Wi-Fi communication interface for transmission to the remote backend. If an abnormal condition is detected, the local controller may also trigger a relay or H-bridge-assisted protective action. Thus, the block diagram represents not only the hardware interconnections but also the functional sequence of sensing, processing, decision making, and communication. The system incorporates two individual flow meters to measure household water usage and a master flow meter to record the total consumption, thereby enabling consistency checks between aggregated and overall usage. Auxiliary infrared (IR) proximity sensors (IR-1 and IR-2) are incorporated to provide contextual activity information near the user access point. These sensors are not used for volumetric flow measurement; instead, they serve as supporting inputs for the rule-based anomaly-checking logic, while the primary leak and consumption analysis remains based on the YF-B1 flow-sensor readings. The microcontroller interfaces with an H-bridge driver that controls two pumps, providing both supply regulation and automated cutoff in case of detected irregularities. A 12 V, 4 Ah rechargeable battery ensures an uninterrupted power supply for reliable operation, while a potentiometer is included for calibration and control adjustments. Additionally, an LCD display provides real-time feedback on flow measurements and billing information, ensuring transparency and user awareness. Overall, this modular configuration promotes ease of maintenance, low deployment cost, and compatibility with different building layouts for fair billing and robust leak detection.

### Hardware components

**Fig. 3:**
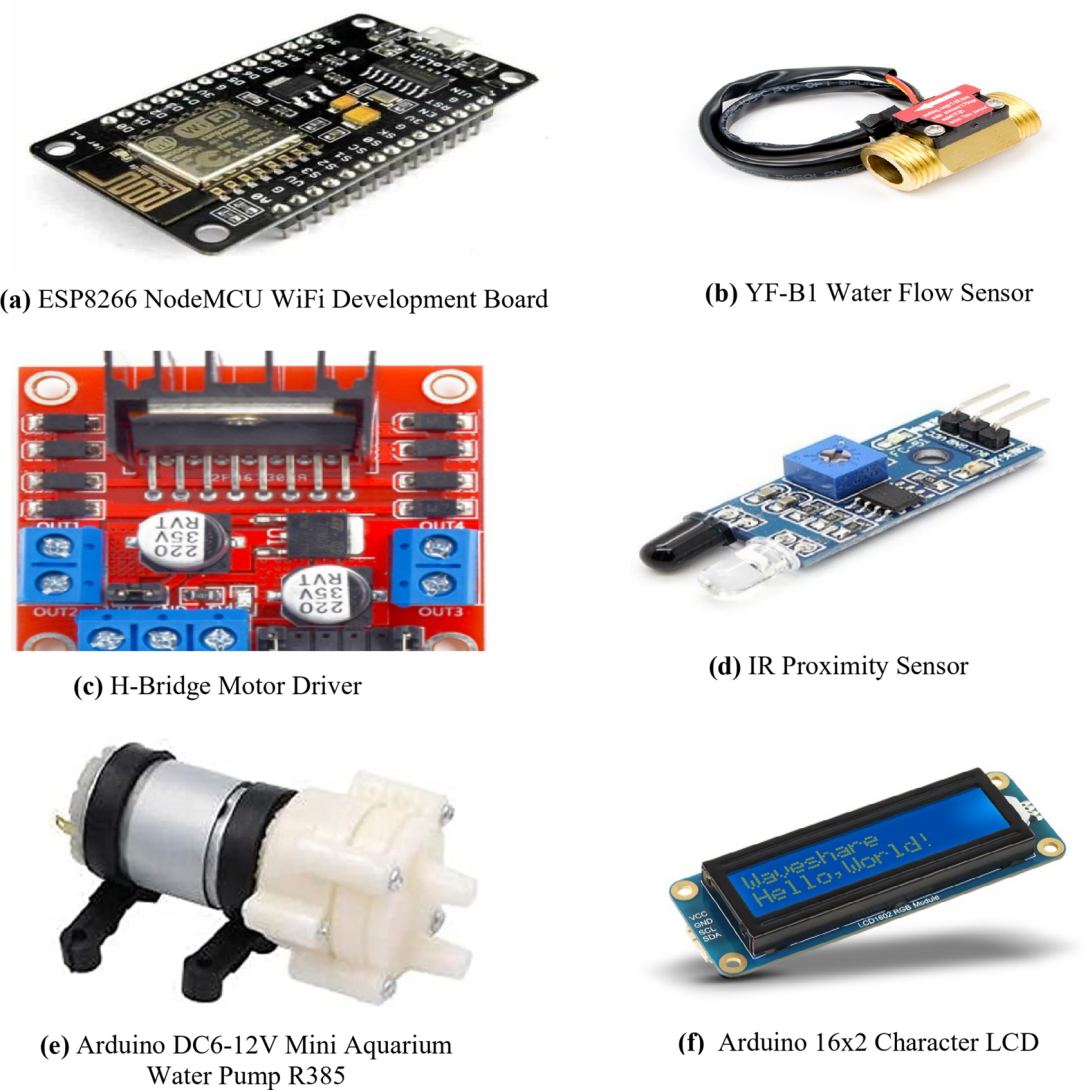
The hardware components of the proposed SWBS.

The SWBS hardware configuration, shown in Fig. 3, integrates the main sensing, control, communication, and power components required for prototype operation. The principal hardware elements are summarized below:

- **ESP8266 NodeMCU**: Serves as the central microcontroller with built-in Wi-Fi capability for data acquisition, processing, and wireless transmission to the server.
- **YF-B1 Water Flow Sensor**: A Hall-effect-based sensor that generates pulses proportional to water volume, enabling precise flow rate computation.
- **H-Bridge Motor Driver**: Controls the DC water pump during simulation and testing scenarios.
- **IR Proximity Sensors** Used as auxiliary contextual sensors near the user interaction zone to indicate local activity during controlled testing. They are not used as water-flow meters; rather, they provide supplementary information that can support the rule-based interpretation of abnormal flow events detected by the YF-B1 sensor.
- **12 V**,** 4 Ah rechargeable battery** Provides backup power for the prototype during offline or low-grid-availability operation. The nominal stored energy is approximately 48 Wh, and the effective runtime depends on the average current demand of the sensing, communication, display, and pump subsystems.
- **DC6–12 V R385 Water Pump**: Simulates various flow conditions for calibration and testing.
- **16 × 2 Character LCD Display**: Presents real-time usage data, billing totals, and error messages to end-users.

For deployment-oriented assessment, the battery runtime can be approximated as: $\:T\approx\:C/{I}_{\mathrm{avg}}$, where $\:T$ is the operating time in hours, $\:C$ is the battery capacity in ampere-hours, and $\:{I}_{\mathrm{avg}}$is the average load current in amperes. For the selected 12 V, 4 Ah battery, the nominal energy storage is approximately 48 Wh. In practice, the achievable runtime depends strongly on the operating mode: during sensing, local processing, and wireless transmission, the power demand is relatively modest, whereas continuous pump actuation significantly increases current draw and reduces runtime. Therefore, the battery in the present prototype is intended primarily as a backup and experimental supply source rather than as a long-duration standalone energy system under continuous pumping conditions.

This modular configuration ensures flexibility, ease of maintenance, and scalability for various deployment environments.

### Software architecture

The firmware of the proposed SWBS was developed in the Arduino IDE and organized into several functional modules, each responsible for a specific sensing, processing, communication, or protection task. These modules are summarized as follows.

1. **Sensor Data Acquisition**: Reads the pulse output from the YF-B1 sensor and converts it into flow rate and cumulative volume using a calibrated conversion factor.
2. **Multi-Stage Calibration & Error Correction**: Relying on a single calibration constant, traditional flow meters often produce significant errors at extreme flow rates. Address this limitation by applying the proposed piecewise calibration algorithm, which dynamically adjusts readings across three flow ranges—low, medium, and high—thereby reducing measurement error to below 3%.
3. **Anomaly Detection**: The system employs a Cumulative Sum (CUSUM) algorithm as a lightweight statistical change-detection method to identify abnormal deviations in water-consumption patterns. Unlike simple threshold-based techniques, CUSUM accumulates small but persistent deviations between the measured flow signal and its expected baseline, allowing the detection of gradual leaks or unusual usage events that may otherwise remain unnoticed. This makes it particularly suitable for embedded IoT platforms, where low computational complexity and real-time responsiveness are essential.

- **CUSUM Algorithm**: Detects gradual or sudden deviations in consumption behavior by monitoring the cumulative departure of the flow signal from its normal operating baseline.
- **Rule-Based Validation**: Interprets abnormal flow events by combining the YF-B1 sensor output with auxiliary IR proximity-sensor states. In this design, the IR signal provides contextual support only and is used to reduce false alarms during controlled operation; the primary indication of leakage remains based on persistent abnormal flow behavior.

4. **Data Transmission**: After local preprocessing, calibration, and anomaly screening, the ESP8266 transmits the processed consumption records and status flags to a remote backend through Wi-Fi communication. The application-layer exchange is implemented using HTTP POST, while the underlying communication relies on the standard TCP/IP protocol stack supported by the ESP8266 network interface. This design enables remote data logging, cloud-side visualization, and IoT-based monitoring without requiring continuous manual data collection.
5. **User interface and alerts**: Updates the LCD with consumption data in real-time and triggers visual/audio alerts when anomalies (e.g., leaks) are detected.
6. **Lock Flow Module**: for over water flow (abnormal consumption) or when preventing charging.

### Control logic

**Fig. 4:**
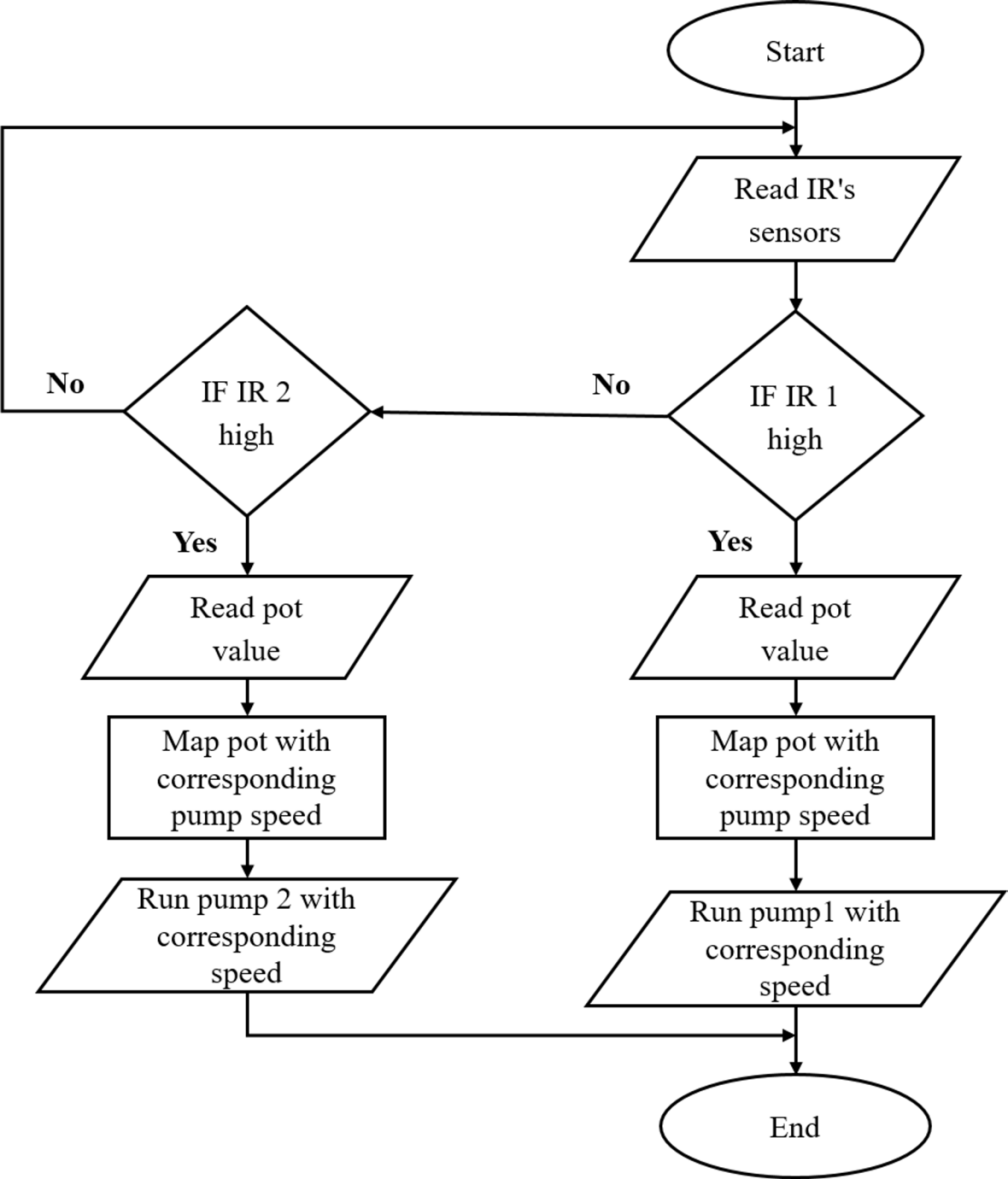
The system model flow chart.

The operational logic of the SWBS follows a structured sequence that governs initialization, sensing, processing, communication, and protective response as shown in Fig. 4. The main steps of this control logic are summarized below:

- Initializing all sensors and establishing Wi-Fi connectivity.
- Flow and IR sensors begin continuous monitoring.
- Raw sensor data are processed locally on the ESP8266 NodeMCU through the multi-stage calibration and anomaly-detection algorithms. The resulting flow estimates, status flags, and alert conditions are then displayed locally on the LCD and transmitted through the Wi-Fi link to the remote server for cloud-connected logging and monitoring.
  - If a leak or anomaly is detected, the system:
  - Activates an alarm.
- Optionally cuts off water supply using the H-Bridge driver.
- Data logs are updated for long-term billing and analytics.

**Fig. 5:**
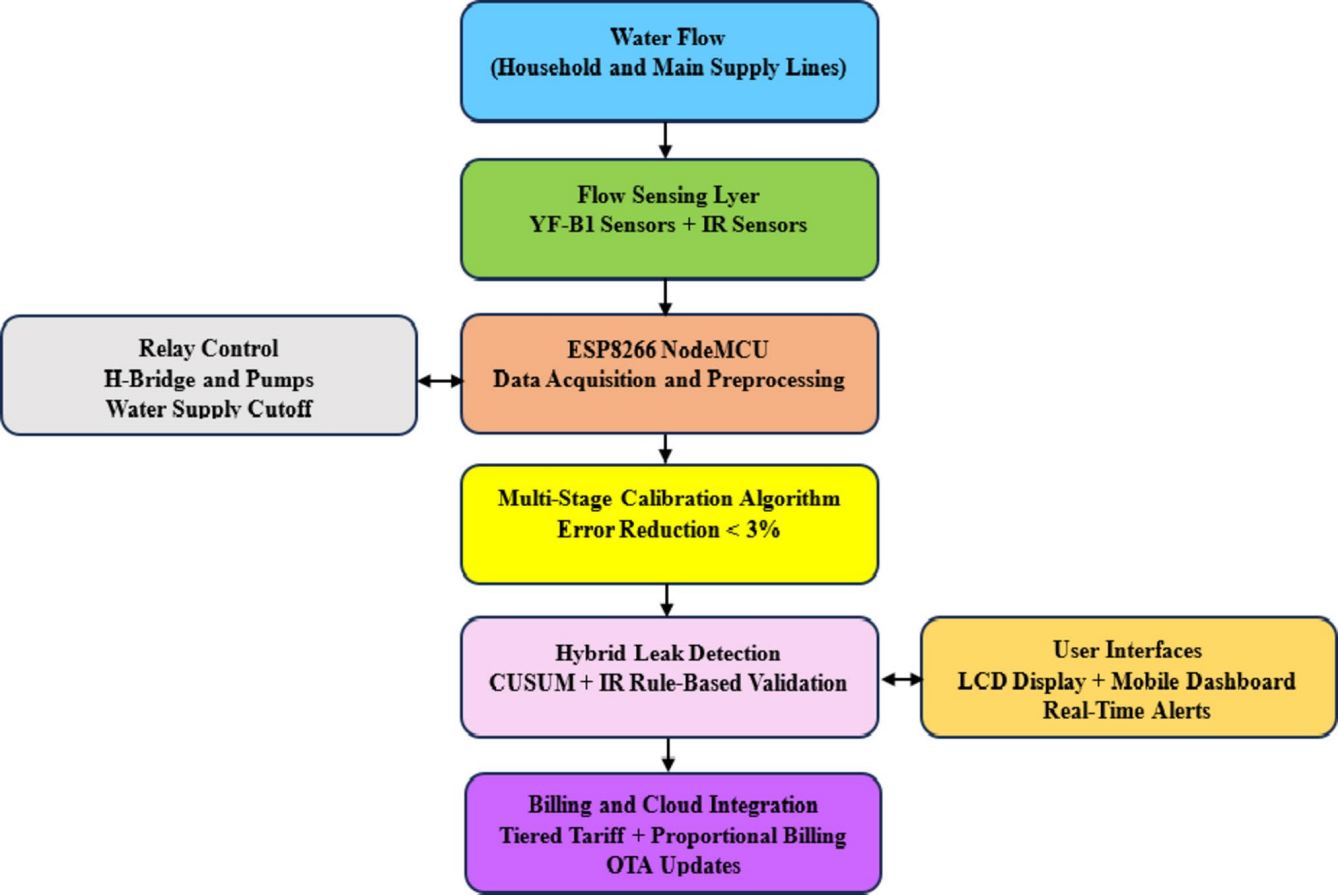
Comprehensive SWBS workflow diagram.

Operationally, the SWBS follows a sequential workflow in which sensing, processing, decision making, and communication are tightly coupled. First, the embedded controller initializes the sensing, display, and communication modules. Next, pulse data from the inline flow sensor are continuously sampled and transformed into raw flow estimates. These raw values are then corrected by the adaptive calibration routine and checked for abnormal patterns using the hybrid detection logic. The validated outputs are displayed locally and transmitted remotely, while any critical anomaly can initiate a local protective response. Figure 5 summarizes this end-to-end workflow from physical water flow to billing-support data generation.

This layered control logic ensures both real-time responsiveness and historical data integrity. Figure 5 illustrates the complete workflow of the SWBS:

1. Water Flow Layer – Represents water moving through both household pipes and the main supply line.
2. Flow Sensing Layer – YF-B1 flow sensors and IR sensors collect real-time usage and activity data.
3. Data Acquisition—The ESP8266 NodeMCU processes raw sensor data for initial filtering.
4. Multi-Stage Calibration – Adaptive algorithm ensures < 3% error across 1–30 L/min flow range.
5. Anomaly Detection—A hybrid approach combining CUSUM statistical analysis with IR rule-based validation to detect leaks and unauthorized usage.
6. Billing and Cloud Integration – Final consumption data are transmitted to a cloud-connected backend for remote logging, monitoring, and billing support. Future versions may incorporate full OTA update management and expanded automated billing-service integration.

Additional modules:

- Relay and Pump Control—H-Bridge drivers manage water cut-off during anomalies.
- User Interfaces— Liquid Crystal Display (LCD) displays and mobile dashboards deliver real-time alerts and reports.

### Integration and communication

The communication layer of the proposed SWBS is based on the built-in Wi-Fi capability of the ESP8266 NodeMCU, which connects the sensing unit to a remote backend through HTTP POST over the TCP/IP communication stack. In the implemented prototype, the backend is used primarily for remote data logging, monitoring, and record storage, whereas the time-critical tasks of pulse acquisition, flow estimation, calibration, anomaly detection, and local protective control are executed on-device. This architecture was intentionally selected to preserve real-time response and local functionality even under unstable internet connectivity.

In the present prototype, cloud interaction is therefore limited to data reception, logging, and monitoring support. More advanced service-layer functions, such as over-the-air (OTA) firmware management, remote tariff synchronization, and fully automated cloud-based billing orchestration, were not experimentally implemented in this study and are considered part of the future development roadmap. For larger-scale deployments, a hybrid edge–cloud architecture can be adopted, where real-time protection and sensing remain local, while computationally heavier analytics, utility-side billing services, and long-term optimization are migrated to cloud infrastructure. For large-scale deployments, the architecture can be expanded through LPWAN (e.g., LoRaWAN) integration, enabling city-level smart metering with minimal infrastructure cost.

Accordingly, the proposed SWBS should be understood as an IoT-based edge-sensing platform in which embedded measurement and control are combined with wireless connectivity and cloud-connected data visibility.

### Mathematical model for sensing, calibration, billing, and anomaly detection

This section presents the mathematical foundation of the proposed Smart Water Billing System (SWBS), linking the physical sensing process to the computational logic used for calibration, anomaly detection, consistency verification, and billing. To improve clarity, the formulation is organized according to the main functional stages of the system: conversion of flow-sensor pulses into flow-rate information, adaptive calibration across varying operating conditions, estimation of cumulative water usage, validation of measurement consistency across multiple meters, detection of anomalous flow behavior, and calculation of the corresponding billing quantities. In the current prototype, these operations are executed locally on the embedded controller in real time, while the remote backend is used mainly for storage and monitoring support.

In the current prototype, the mathematical operations associated with sensor transduction, adaptive calibration, anomaly detection, and billing-variable estimation are executed locally on the embedded controller in real time. The cloud-connected backend is not used for primary decision making in the present implementation; rather, it serves as a remote record and monitoring layer.

#### Sensor Transduction Model

The YF-B1 Hall-effect turbine flow sensor produces a sequence of pulses proportional to the volumetric flow. Over a sampling interval Δ*t* [s], the pulse frequency *f*(t) [Hz] and raw flow rate *Q*_raw_(t) [L/min] are given by:

The first step in the SWBS processing chain is the conversion of the pulse output generated by the YF-B1 Hall-effect flow sensor into a physically meaningful flow-rate estimate. Because the sensor produces pulses whose frequency is proportional to the water flow passing through the turbine rotor, the embedded controller can estimate the instantaneous flow rate from the pulse count observed over a known sampling interval.

1 $$\:f\left(t\right)=\frac{{\Delta\:}\mathrm{N}}{{\Delta\:}\mathrm{t}}$$

2 $$\:{Q}_{raw}\left(t\right)=60\:{v}_{p}\:f\left(t\right)$$

Here, $\:{\Delta\:}N$ is the number of pulses counted during the sampling interval $\:{\Delta\:}t$, $\:f\left(t\right)$ is the resulting pulse frequency, $\:{v}_{p}$ is the calibration constant expressed in liters per pulse, and $\:{Q}_{\mathrm{raw}}\left(t\right)$ is the uncalibrated instantaneous flow rate. This formulation provides the raw sensing output that is subsequently corrected by the adaptive calibration stage.

#### Calibration model

The calibration constant $\:{v}_{p}$ is determined experimentally by comparing the measured pulse count with a reference volume:

Because low-cost Hall-effect flow sensors may exhibit flow-rate-dependent nonlinearity, a calibration stage is required to convert the raw pulse-based estimate into a more accurate measurement. In the proposed SWBS, calibration is performed experimentally by comparing the pulse count with a known reference volume and then applying a piecewise correction strategy across different flow ranges.

3 $$\:{\widehat{v}}_{p}=\:{median}_{k}\left(\frac{{V}_{ref,k}}{{N}_{k}}\right)$$

Here, $\:{V}_{\mathrm{ref},k}$ is the known reference volume used in calibration trial $\:k$, $\:{N}_{k}$ is the corresponding pulse count, and $\:{\widehat{v}}_{p}$ is the calibrated liters-per-pulse constant estimated from the median of the calibration trials. This value provides a robust baseline conversion factor that is less sensitive to outlier trials.

4 $$\:c\left(Q\right)=\left\{\begin{array}{c}{\alpha\:}_{1}Q+{\beta\:}_{1},\:\:\:\:\:\:\:\:\:\:\:\:\:\:\:\:\:\:\:\:\:\:\:\:\:\:\:Q\in\:\left[1,\:5\right]\:L/min,\:\:\:\\\:{\alpha\:}_{2}Q+{\beta\:}_{2},\:\:\:\:\:\:\:\:\:\:\:\:\:\:\:\:\:\:\:\:\:\:\:\:\:\:\:Q\in\:\left[5,\:15\right]\:L/min,\:\:\:\\\:{\alpha\:}_{3}Q+{\beta\:}_{3},\:\:\:\:\:\:\:\:\:\:\:\:\:\:\:\:\:\:\:\:\:\:\:\:Q\in\:\left[15,\:30\right]\:L/min.\:\:\end{array}\right.$$

The compensated flow and cumulative volume are:

5 $$\:Q\left(t\right)={Q}_{raw}\left(t\right)c\left({Q}_{raw}\left(t\right)\right)$$

6 $$\:V\left(t\right)=\sum\:_{r\le\:t}\frac{Q\left(t\right)}{60}\:{\Delta\:}t$$

In this formulation, $\:{\alpha\:}_{i}$ and $\:{\beta\:}_{i}$ are the calibration coefficients associated with flow range $\:i$, $\:Q\left(t\right)$ is the calibrated instantaneous flow rate, and $\:V\left(t\right)$ is the cumulative water consumption obtained by integrating the corrected flow over time. This adaptive formulation is central to the proposed system because it reduces the sensor nonlinearity observed under different flow conditions.

#### Accuracy and error estimation

To quantify the accuracy of the calibrated measurements, the estimated water volume is compared with the corresponding reference volume obtained from controlled experimental trials. The resulting relative error is used to evaluate whether the calibration procedure satisfies the accuracy target required for fair billing and reliable anomaly detection.

7 $$\:{\epsilon\:}_{k}=\frac{{V}_{k}-{V}_{ref,k}}{{V}_{ref,k}}$$

Here, $\:{V}_{k}$ is the measured water volume in trial $\:k$, $\:{V}_{\mathrm{ref},k}$ is the reference volume, and $\:{\epsilon\:}_{k}$ is the corresponding relative error. The SWBS is designed to operate with a target error below 3%, which provides a practical balance between low-cost sensing and billing-grade measurement reliability.

#### Multi-meter consistency

When multiple meters (F1, F2) are connected to the same main line, the total consumption is validated against the main meter. In multi-user building deployments, per-unit metering must remain consistent with the total water entering the building. For this reason, the SWBS includes a consistency-validation step in which the sum of the household branch-meter volumes is compared against the main meter reading. This comparison is important for fairness, system validation, and the detection of possible discrepancies caused by leakage or measurement drift.

8 $$\:{V}_{M}\left(t\right)\approx\:{V}_{F1}\left(t\right)+{V}_{F2}\left(t\right)$$

9 $$\:{\updelta\:}\left(t\right)={V}_{M}\left(t\right)-{V}_{F1}\left(t\right)-{V}_{F2}\left(t\right)$$

Here, $\:{V}_{M}\left(t\right)$ is the cumulative volume measured by the main meter, $\:{V}_{F1}\left(t\right)$ and $\:{V}_{F2}\left(t\right)$ are the cumulative volumes of the individual branch meters, and $\:\delta\:\left(t\right)$ represents the reconciliation discrepancy. In normal operation, this discrepancy is expected to remain small, thereby confirming consistency between distributed user-level measurements and the total building-level consumption.

#### Anomaly and leak detection

To detect leakage and abnormal consumption events, the proposed SWBS combines a statistical change-detection method with rule-based physical validation. The statistical component is based on the Cumulative Sum (CUSUM) technique, which is widely used for detecting small but persistent shifts in monitored signals. In the context of the SWBS, CUSUM tracks the cumulative deviation of the calibrated flow rate from a moving baseline, enabling the early detection of flow anomalies that may indicate leakage, abnormal usage, or unauthorized consumption. Because of its low computational burden and suitability for sequential monitoring, CUSUM is particularly appropriate for implementation on embedded microcontroller platforms such as the ESP8266 NodeMCU. Two methods are applied:

**(a) CUSUM-based detection**:

10 $$\:{S}_{t}=\mathrm{max}\left\{0,\:{S}_{t-1}+\left(Q\left(t\right)-{m}_{t}\right)-v\right\},\:\:\:\:\:\:\:\:alarm\:if\:{S}_{t}\ge\:h$$

Here, $\:{m}_{t}$ is the moving median baseline representing normal flow behavior, *v* is the drift parameter used to suppress minor random fluctuations, and *h* is the decision threshold beyond which an alarm is triggered. In practice, this formulation allows the system to distinguish persistent abnormal flow from ordinary short-term fluctuations, thereby improving leak-detection reliability.

**(b) Rule-based detection**:

In the proposed SWBS, leakage assessment is driven primarily by the flow continuity and abnormality pattern measured by the YF-B1 sensor. The auxiliary IR proximity sensor does not measure water flow and is not treated as a standalone human-presence detector for definitive leak classification. Instead, its signal is used only as a contextual indicator during controlled operation, helping the system distinguish between continuous unattended flow and user-associated activity in the rule-based validation stage. A suspected leak condition is reinforced when the measured flow persists beyond a minimum duration threshold while no corresponding auxiliary activity indication is detected by the IR proximity sensor during the observation window.

11 $$\:Leak\:if\:Q\left(t\right)>0\:for\:duration\ge\:{\tau\:}_{min},\:\:\:\:\:and\:IR=0\:$$

where $\:\theta\:\:$is the leak-threshold flow, $\:{\tau\:}_{min}$is the minimum persistence time, and IR = 0 denotes the absence of auxiliary activity indication from the infrared proximity sensor. This criterion is used only as a supporting decision rule and does not replace the primary flow-based leakage assessment.

Together, the statistical CUSUM detector and the rule-based contextual check form a hybrid anomaly-detection mechanism that is suitable for real-time implementation and capable of reducing false alarms while preserving early leak sensitivity.

#### Billing model

Two billing strategies are supported. Once water consumption has been estimated and validated, the SWBS translates the measured usage into billing quantities. To accommodate different deployment scenarios, the system supports both a tiered tariff framework, where higher consumption may correspond to different pricing blocks, and a proportional cost-sharing framework, which is particularly relevant in multi-unit buildings supplied through a common bill.

**(a) Tiered Tariff**:

12 $$\:{C}_{i}=\sum\:_{j=1}^{J}{p}_{j}.\mathrm{min}\left\{{max}\left({V}_{i}-\sum\:_{l<j}{b}_{l},0\right),{b}_{j}\right\}$$

where $\:{b}_{j}$ is the block size of tier *j* and $\:{p}_{j}$ is the price per liter in tier *j.*

**(b) Proportional Cost Sharing**:

13 $$\:{C}_{i}=B.\frac{{V}_{i}}{{\sum\:}_{u=1}^{U}{V}_{u}}$$

where *B* is the total bill for the building, $\:{V}_{i}$ is the volume for unit *i*, and *U* is the total number of units.

**Consistency check**:

14 $$\:\left|{V}_{M}-\sum\:_{i}{V}_{i}\right|<\eta\:{V}_{M}$$

where *η* is the tolerance (e.g., 3%).

In the tiered-tariff model, $\:{b}_{j}$ denotes the block size of tariff tier $\:j$ and $\:{p}_{j}$ is the corresponding unit price. In the proportional-sharing model, $\:B$is the total building-level bill, $\:{V}_{i}$is the consumption of unit $\:i$, and $\:U$ is the total number of units. The consistency condition in Eq. (14) ensures that the building-level total remains compatible with the sum of the individual billed volumes within an acceptable tolerance $\:\eta\:$. This formulation supports fair billing while preserving traceability between metered consumption and the final cost allocation.

Overall, the mathematical formulation of the SWBS provides a unified framework that links raw sensor measurements, adaptive correction, anomaly identification, consistency checking, and billing computation. This integrated formulation supports the practical implementation of the prototype and ensures that the sensing, monitoring, and billing functions are mathematically consistent with the physical operation of the system.

## System implementation and testing

The implementation and testing phase of the SWBS was carried out to validate the functional integration of the hardware and software components and to evaluate the accuracy and reliability of the proposed design under controlled operating conditions. The results reported in this study were obtained from a physical laboratory prototype rather than from a purely simulated dataset. This stage therefore involved assembling the prototype, configuring the firmware, generating controlled water-flow conditions, and recording real-time pulse-based measurements in order to assess baseline sensor behavior, calibration performance, anomaly response, and multi-meter consistency.

### Prototype assembly

The assembled prototype incorporated the ESP8266 NodeMCU as the main control, processing, and communication unit, connected to inline YF-B1 flow sensor(s) for water-consumption monitoring. During laboratory testing, controlled flow conditions were produced using a DC pump and H-bridge driver, while a 2-liter reference container was used as the known ground-truth volume for calibration and baseline validation. Supporting modules, including a 12 V, 4 Ah rechargeable battery, IR proximity sensors, and an LCD interface, were integrated to provide backup power, contextual sensing, and real-time user feedback. In the physical arrangement, all measured water passed through the YF-B1 sensor before being collected or routed to the outlet, ensuring that the recorded pulse stream corresponded directly to the tested flow event.

### Firmware deployment

The system firmware was developed in the Arduino IDE using C + + and uploaded to the NodeMCU. The code modules handled pulse acquisition from the YF-B1 sensors, conversion of pulses into volumetric flow data, and application of correction factors derived from experimental calibration. In addition, the firmware enabled IoT-based wireless data transmission from the ESP8266 NodeMCU to a remote server and updated the LCD display in real time. This firmware layer therefore combines embedded sensing with networked communication, allowing the prototype to operate as a connected smart-metering node rather than as a standalone local instrument. The firmware execution sequence was guided by the operational flowchart shown in Fig. 4, which illustrates the initialization, monitoring, calibration, anomaly-checking, error-handling, and alert-generation steps carried out during system operation. In practical operation, the firmware follows a fixed execution cycle. First, it initializes the sensor interfaces, LCD module, and Wi-Fi communication stack. It then counts the pulses produced by the YF-B1 flow sensor over the selected sampling interval and converts them into raw flow estimates. These values are processed through the adaptive calibration routine and then passed to the anomaly-detection module, which evaluates both statistical deviations and contextual rule-based conditions. Finally, the resulting measurements and status flags are displayed locally and transmitted to the remote backend. This implementation sequence corresponds to the operational flowchart presented in Fig. 4.

### Testing methodology

The experimental data used in this study were acquired in real time from the physical SWBS prototype under controlled laboratory conditions. Each test involved generating a known water-flow event, recording the corresponding sensor pulse counts through the embedded controller, and comparing the estimated values either with a known reference container volume or with the main/reference flow meter, depending on the test objective. Accordingly, the four tests were designed not merely as isolated demonstrations, but as sequential validation stages moving from raw-sensor characterization to integrated multi-meter verification.

To rigorously evaluate system performance, four experimental tests were designed. Each test simulated practical usage conditions and verified the system’s capacity to maintain accuracy across variable flow conditions:

- **Test (1): Baseline accuracy**—A 2-liter standard container was filled under multiple flow conditions (low, medium, high, and intermittent). Pulse counts were recorded and compared with the actual measured volume to derive error equations.
- **Test (2): Flow velocity calibration**—Water was passed at varying speeds to determine the relationship between pulse frequency and flow velocity. From these trials, the average amount of water per pulse was calculated, and correction factors were refined.
- **Test (3): Multi-range error correction** — A calibration table was developed across different flow ranges (1–30 L/min), addressing the variability of error at low and high flow rates. This adaptive correction significantly improved measurement accuracy compared with using a single constant.
- **Test (4): Integrated system validation**—Flow Meter 1, Flow Meter 2, and the main reference flow meter were tested in three scenarios: (i) Flow Meter 1 only, (ii) Flow Meter 2 only, and (iii) both flow meters combined. The results confirmed that individual meters closely matched the main reference, while their combined readings maintained negligible error, validating the scalability of the system for multi-unit applications.

### Implementation outcomes

The implementation and testing phase confirmed that the SWBS operates effectively as a low-cost, IoT-enabled water monitoring and billing system. In particular, the combination of embedded sensing, Wi-Fi communication, and remote data logging demonstrated the practical feasibility of the proposed IoT architecture for real-time consumption visibility and distributed monitoring. The integration of hardware and firmware was seamless, and the system achieved high accuracy once calibration factors were applied. The ability to combine multiple flow meters without significant error highlights its suitability for larger-scale deployments such as apartment buildings or community networks. Furthermore, the real-time data reporting and LCD feedback demonstrated practical usability for both consumers and utility providers.

**Fig. 6:**
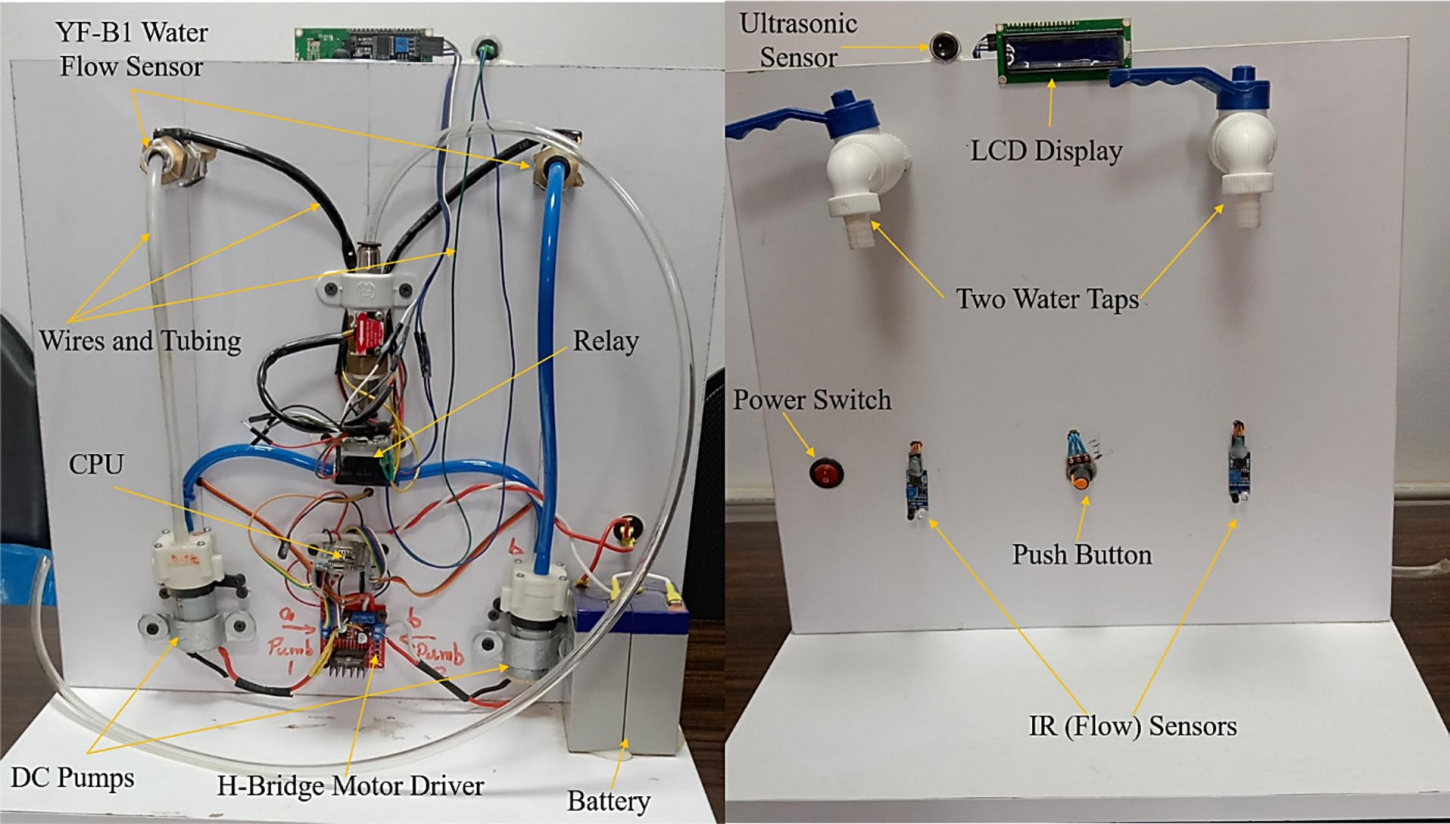
The practical hardware prototype of the proposed SWBS.

Figure 6 shows the practical hardware prototype of the proposed Smart Water Billing System (SWBS), which integrates sensing, control, and communication modules into a functional setup. The system is powered by a 12 V, 4 Ah rechargeable battery that ensures continuous operation. Two DC pumps deliver water to dedicated taps, with their operation controlled by an H-bridge motor driver and relay module, enabling safe and efficient switching of higher current loads. The NodeMCU ESP8266 microcontroller serves as the central processing unit, acquiring signals from sensors, executing billing logic, and controlling pumps, relays, and the LCD display. Flow sensing is achieved primarily through the YF-B1 Hall-effect water flow sensor which is installed inline within the branch water path of the prototype, which measures consumption by converting turbine rotor motion into proportional electrical pulses. These pulses are processed by the microcontroller to calculate instantaneous flow rate (L/min) and total water usage (liters), which in turn forms the basis for accurate billing. Additional IR proximity sensors are included as auxiliary contextual sensors near the user-side access points to support controlled activity interpretation during anomaly detection. These sensors are not used to measure water flow directly; volumetric flow measurement is performed exclusively by the YF-B1 Hall-effect flow sensor, while the ultrasonic sensor is used for water-level measurement. A push button and power switch allow manual system control, and an LCD display gives real-time feedback on consumption and billing information. Supporting components such as wiring and tubing complete the setup, ensuring proper integration of mechanical flow control with electronic sensing. Collectively, this prototype validates the conceptual hardware block diagram, demonstrates efficient and user-friendly operation, and serves as the experimental platform for the performance evaluation presented in Sect. 4.

From a deployment perspective, the selected battery capacity is sufficient for short-term backup and controlled prototype operation; however, long-duration operation under continuous pump duty would require a larger energy source or a redesigned low-power actuation strategy.

## Results and discussion

This section presents and analyzes the outcomes of the experimental validation performed on the physical SWBS prototype. The reported results were obtained from real-time pulse-based measurements collected during controlled laboratory tests and were used to evaluate sensor behavior, calibration effectiveness, integrated multi-meter consistency, and anomaly-detection performance. Together, the presented results show how the proposed SWBS evolves from a raw pulse-based sensing unit into a calibrated and validated platform capable of supporting accurate water-consumption monitoring and fair billing.

### Flow meter test (1)

In the first test, we conducted ten separate trials to check the accuracy of the flow meter by filling a 2-liter standard container under different flow conditions. To simulate real-world usage, the faucet was opened at high, medium, and low speeds, and in some cases, it was intermittently opened and closed while filling. During each trial, the pulses generated by the flow meter were carefully recorded. These pulses were then converted into corresponding water volumes and compared with the actual 2-liter standard to see how close the measurements were. By analyzing the differences, we were able to calculate the error rate for each trial. From these results, we derived an equation that can be used to more accurately estimate the amount of water in any standard container during future measurements. The complete results of Flow Meter Test (1), including the recorded pulses, calculated volumes, and error rates at the different flow speeds, are presented in Table 3.

**Table 3:** Results of Flow Meter Test (1) under varying flow conditions.

Test No.	Time (s)	Pulse Count	Pulse Rate (pulse/s)	Calculated Volume (L)	Reference Volume (L)	Error (L)	Error (%)	Flow Condition
1	7.07	1381	195.33	1.94	2.00	−0.06	3.00%	High
2	7.71	1161	150.58	1.89	2.00	−0.11	5.50%	High
3	8.28	1242	150.00	1.96	2.00	−0.04	2.00%	Medium
4	8.99	1121	124.69	1.87	2.00	−0.13	6.50%	Medium
5	16.27	1202	73.88	2.02	2.00	+ 0.02	1.00%	Medium
6	17.92	1154	64.40	1.98	2.00	−0.02	1.00%	Low
7	23.26	1090	46.86	1.93	2.00	−0.07	3.50%	Low
8	26.53	1065	40.14	1.91	2.00	−0.09	4.50%	Low
9	55.75	881	15.80	1.85	2.00	−0.15	7.50%	Very Low
10	128.35	447	3.48	1.78	2.00	−0.22	11.00%	Very Low

The calculated volume is derived from the recorded pulse count using the sensor’s conversion factor, while the reference volume corresponds to the fixed 2-liter container used during the experiments. The measurement error in liters is obtained as the difference between the calculated volume and the reference volume, and the error percentage is computed by normalizing this difference with respect to the reference volume. The flow conditions (high, medium, and low) are determined based on the level of faucet opening during each test.

These results demonstrate the baseline variability of the uncalibrated sensor response and justify the need for subsequent correction and calibration stages.

### Flow meter test (2)

In the second test, the 2-liter container was filled ten times while deliberately varying the flow speeds by manually adjusting the faucet. The purpose of this test was to determine the error rate of the flow meter at different velocities and to quantify the amount of water corresponding to each pulse generated by the Hall-effect sensor inside the device. For every trial, the number of pulses was recorded, and the volume of water per pulse was calculated by dividing the fixed 2000 ml (the actual capacity of the container) by the pulse count of that trial. From these calculations, it was observed that there is a direct relationship between flow velocity and the number of pulses, where higher flow speeds resulted in higher pulse frequencies. To improve measurement accuracy, the values obtained from the ten trials were averaged to determine an error correction factor. This correction factor was then incorporated into the flow meter’s equation provided in its datasheet to adjust the readings at different flow speeds. The detailed results of Flow Meter Test (2), including the recorded pulse counts, calculated water per pulse, and the derived correction factor, are presented in Table 4.

**Table 4:** Results of Flow Meter Test (2) showing pulse frequency, flow velocity, and water per pulse.

No.	Pulse	Time (sec)	Amount (ml) per pulse	Frequency	Liter/Hour	Liter/Min	Test
1	881	55.75	2.27014756	15.80269	129.147982	2.15246636	1.80150672
2	1090	23.26	1.834862385	46.86156	309.544282	5.15907136	5.34221840
3	1121	8.99	1.78412132	124.6941	800.889877	13.3481646	14.2151279
4	1065	26.53	1.877934272	40.143234	271.390878	4.52318130	4.57632868
5	447	128.35	4.474272931	3.4826646	56.0966108	0.93494351	0.39702376
6	1242	8.28	1.610305958	150	869.565217	14.4927536	17.1
7	1161	7.71	1.722652885	150.58366	933.852140	15.5642023	17.1665369
8	1381	7.07	1.448225923	195.33239	1018.38755	16.9731258	22.2678925
9	1202	16.27	1.663893511	73.878304	442.532268	7.3755378	**-**
10	1154	17.92	1.733102253	64.397321	401.785714	6.69642857	**-**

This test demonstrates that the pulse-to-volume relationship is flow dependent, supporting the need for a more refined calibration strategy than a single fixed conversion constant.

### Flow meter test (3)

**Fig. 7:**
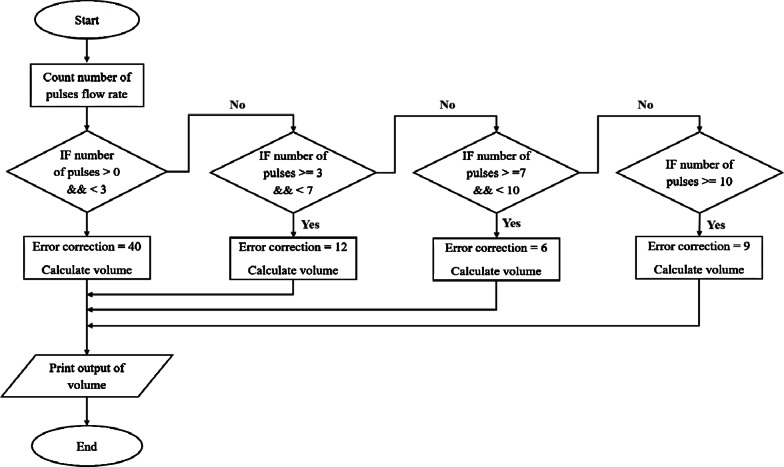
The flowchart of Test (3).

Building on the results from Tests (1) and (2), the third test focused on addressing the issue of varying error rates at different flow speeds. Initially, we noticed that when the flow meter was calibrated for a specific flow rate, such as 1–2 L, the measurements were accurate. However, once we increased or decreased the flow rate from that set value, the readings became inaccurate, with an error margin of ± 5%, as stated in the datasheet. Upon further testing, we discovered that the actual error rate varied depending on the flow rate—for instance, 1 L and 2 L each had different correction factors. This revealed that a single correction factor was not sufficient for all flow rates. To resolve this, we decided to create an error correction factor for each flow range within the sensor’s operating range of 1–30 L per minute. By dividing the flow range into smaller segments and calculating a specific error correction for each, we were able to significantly improve the accuracy of the flow meter across all tested flow rates. The detailed methodology for this adaptive correction approach is illustrated in the flowchart of Fig. 7, which outlines the steps taken to determine and apply error corrections for various flow ranges in Test (3).

The outcome of this test demonstrates that adaptive range-based correction is necessary to maintain acceptable accuracy across the full operating range of the sensor.

### Flow meter test (4)

**Fig. 8:**
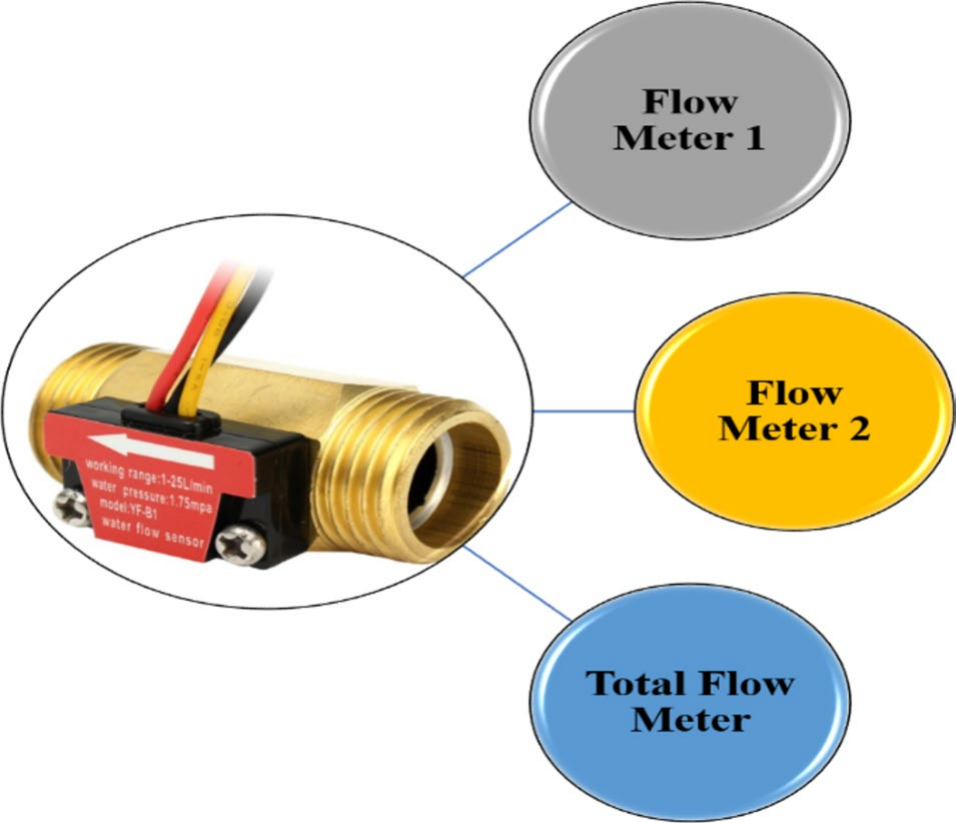
Diagram illustrating the final evaluation of the complete integrated system across three stages.

The final validation stage assessed the complete integrated SWBS under three operating scenarios: Flow Meter 1 only, Flow Meter 2 only, and simultaneous combined operation. These scenarios, illustrated in Fig. 8, were used to verify agreement between branch-level metering and the main reference measurement.

These results demonstrate that the distributed metering architecture remains consistent with the main reference measurement and can therefore support fair per-unit billing in a multi-user configuration.

#### Flow meter (1)

**Fig. 9:**
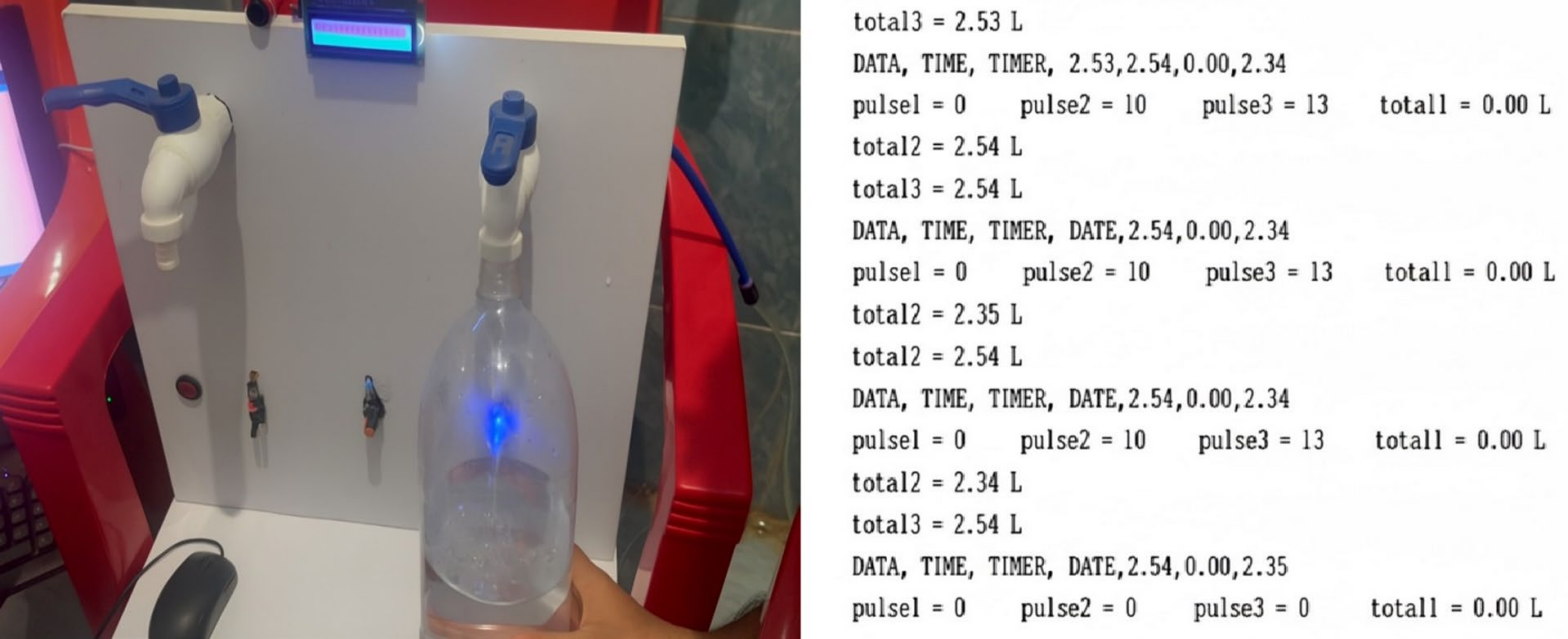
The setup and results of Flow Meter (1) in Test (4).

In the first stage of Test (4), water was supplied through the first flow meter only, and its readings were compared directly with those of the main flow meter. The comparison showed that the calculations closely matched, with only a very small margin of error. This confirmed the reliability of the first flow meter in measuring water flow with accuracy comparable to the main system. Figure 9 illustrates the setup and results of Flow Meter (1) in Test (4), highlighting the consistency between the two meters and the negligible error observed.

#### Flow meter (2)

**Fig. 10:**
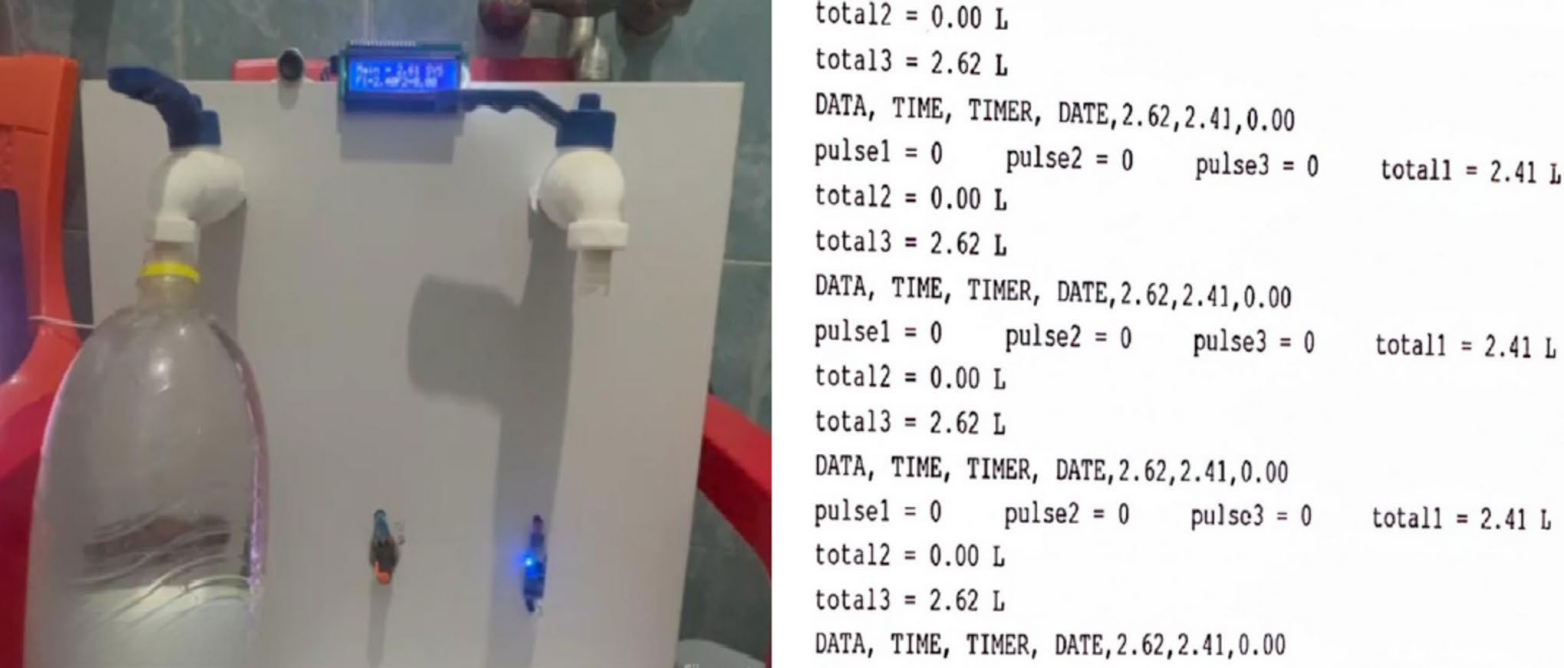
The setup and results of Flow Meter (2) in Test (4).

In the second stage of Test (4), the procedure was repeated using Flow Meter (2), which was connected independently to the household water supply. Water was passed through this flow meter only, and the output was measured, displayed on the monitor, and compared with the readings of the main flow meter. Similar to the first stage, the results showed close agreement between the two measurements, confirming the reliability and accuracy of Flow Meter (2). Figure 10 presents the setup and results of this stage, illustrating the consistency between Flow Meter (2) and the main flow meter.

#### Total flow meter

**Fig. 11:**
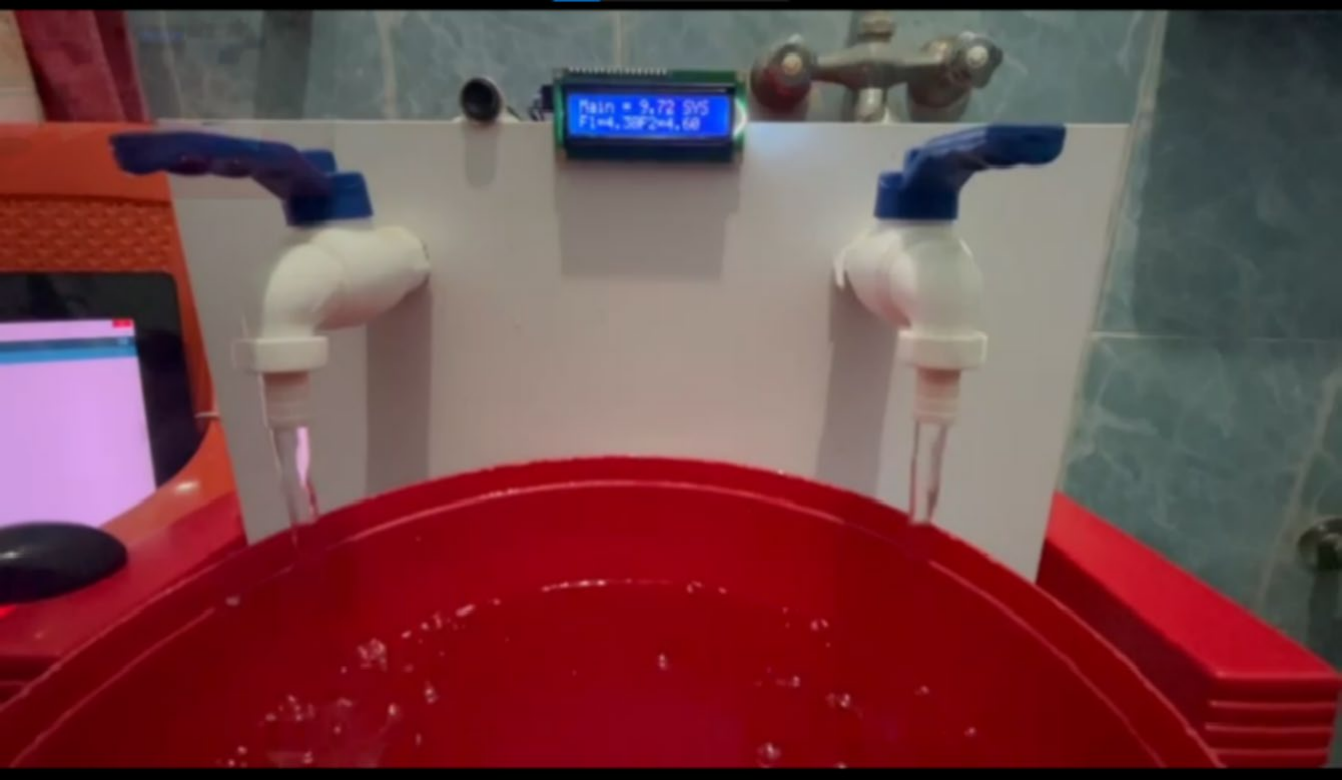
The combined Total Flow Meter test.

In the third stage of Test (4), Flow Meter (1) and Flow Meter (2) were connected simultaneously to the household water supply. Water was passed through both meters, and their individual readings were displayed on the monitor and then summed to obtain the total measured output. This combined value was compared with the reading of the main flow meter using larger standard volumes for validation. The results showed a strong agreement, as the total from Flow Meters (1) and (2) closely matched the output of the main meter with only minimal discrepancy. These findings confirmed the accuracy and reliability of the system when both meters were used together. Figure 11 illustrates the setup of the combined flow meter test, while Table 5 summarizes the results, highlighting the consistency between the individual meters and the main flow meter.

**Table 5:** Comparison of main flow meter with Flow Meter 1 and Flow Meter 2 in the Total Flow Meter test.

Connect using “PLX-DAQ Simple Test”	Data	Main	F1	F2
6:38:22 AM	7/3/2025	0	0	0
6:38:29 AM	7/3/2025	0.57	0.24	0.26
6:38:29 AM	7/3/2025	0.6	0.25	0.27
6:38:40 AM	7/3/2025	1.86	0.82	0.87
6:38:42 AM	7/3/2025	2.14	0.95	1
6:38:56 AM	7/3/2025	3.76	1.69	1.78
6:39:00 AM	7/3/2025	4.15	1.87	1.97
6:39:23 AM	7/3/2025	6.81	3.07	3.25
6:39:25 AM	7/3/2025	7.07	3.19	3.37
6:39:27 AM	7/3/2025	7.33	3.31	3.5
6:39:36 AM	7/3/2025	8.33	3.76	3.98
6:39:42 AM	7/3/2025	9.11	4.12	4.36
6:39:50 AM	7/3/2025	10	4.52	4.79
6:39:52 AM	7/3/2025	10.17	4.6	4.87

### Discussion

This section presents the performance evaluation of the proposed SWBS prototype through controlled experiments designed to assess its accuracy, response time, and practical deployment challenges. Four experimental tests were carried out, as detailed before, and the results were analyzed using statistical performance metrics.

#### Statistical accuracy metrics

To evaluate the accuracy of the SWBS against reference measurements, three standard statistical metrics were used:

**(a) Mean Absolute Error (MAE)**

15 $$\:MAE=\frac{1}{n}\sum\:_{i=1}^{n}\left|{V}_{predicted}-{V}_{refrence}\right|\:$$

Where $\:{V}_{predicted}$ is the cumulative volume measured by the SWBS, $\:{V}_{refrence}$ is the actual volume measured by a calibrated reference container, and *n* is the number of samples. The MAE for the calibrated system was 0.056 L, significantly lower than the uncalibrated YF-B1 sensor, which had an MAE of 0.182 L. This indicates a 69.2% improvement in absolute measurement accuracy after applying the multi-stage calibration.

**(b) Root Mean Square Error (RMSE)**

16 $$\:RMSE=\sqrt{\frac{1}{n}\sum\:_{i=1}^{n}{\left({V}_{predicted}-{V}_{refrence}\right)}^{2}}$$

The calibrated SWBS achieved an RMSE of 0.073 L, while the uncalibrated YF-B1 sensor showed an RMSE of 0.205 L. The reduction in RMSE demonstrates the system’s ability to minimize large deviations across varying flow rates.

**(c) R² Correlation Coefficient**

17 $$\:{R}^{2}=1-\frac{\sum\:_{i=1}^{n}{\left({V}_{refrence}-{V}_{predicted}\right)}^{2}}{\sum\:_{i=1}^{n}{\left({V}_{refrence}-{\stackrel{-}{V}}_{refrence}\right)}^{2}}$$

Where $\:{\stackrel{-}{V}}_{refrence}$ is the mean of reference values. The SWBS achieved an R² value of 0.992, indicating an extremely strong correlation between the calibrated readings and the actual reference values. In contrast, the uncalibrated YF-B1 sensor achieved an R² of 0.945, showing that calibration significantly enhances linearity.

Taken together, these metrics show that the proposed calibration strategy improves both absolute accuracy and measurement linearity, with the calibrated SWBS reducing MAE from 0.182 L to 0.056 L, reducing RMSE from 0.205 L to 0.073 L, and increasing the R² correlation from 0.945 to 0.992 relative to the uncalibrated sensor.

#### Accuracy Comparison with Uncalibrated YF-B1

**Fig. 12:**
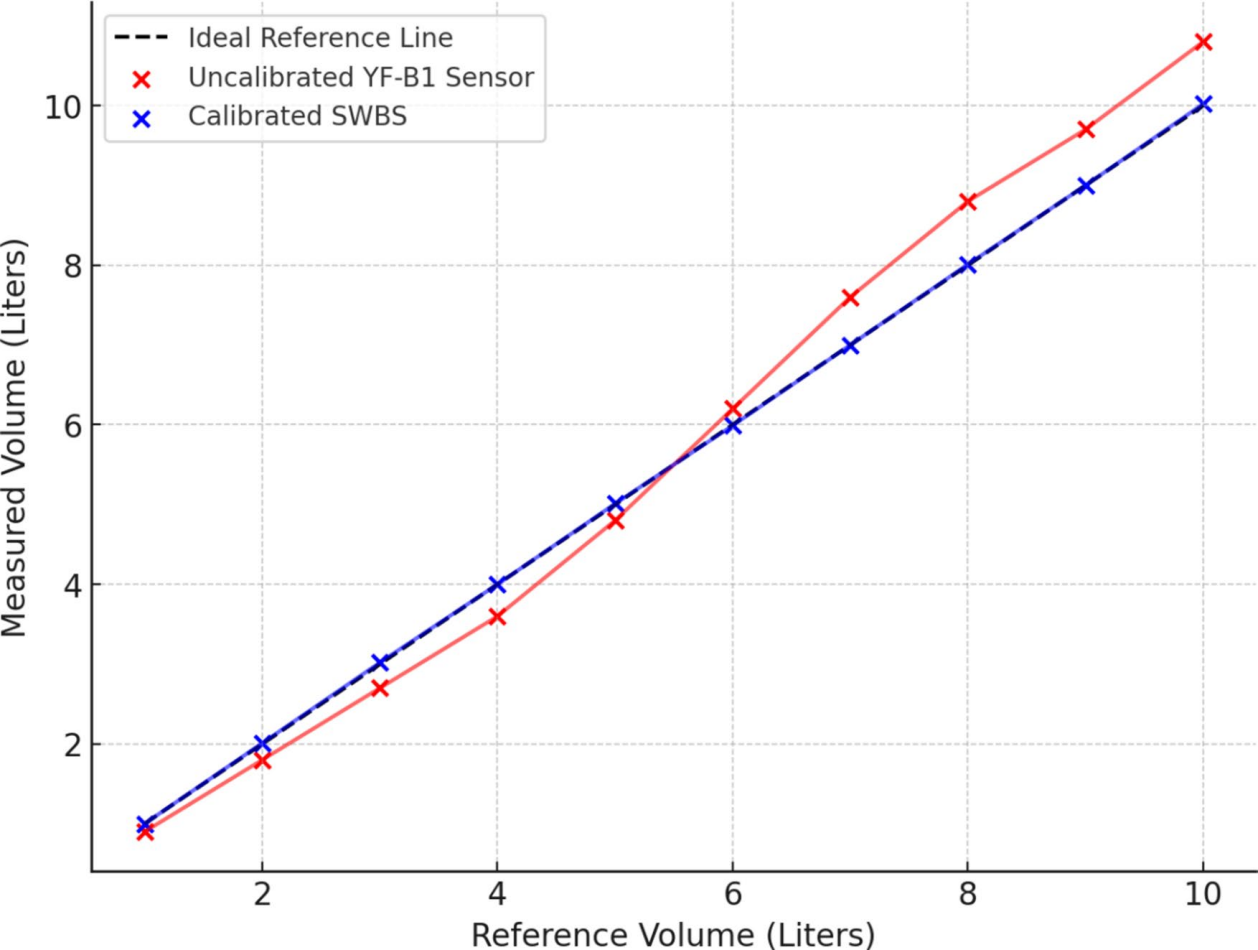
Accuracy Comparison Calibrated SWBS vs. Uncalibrated YF-B1 Sensor.

To highlight the benefit of the proposed multi-stage calibration, a direct comparison was conducted between the calibrated SWBS and the raw YF-B1 readings. Figure 12 shows a scatter plot comparing measured volume against actual reference volume for both configurations:

- **Uncalibrated YF-B1 (red line)**: Divergence at low (< 2 L/min) and high (> 20 L/min) flow rates reflects the nonlinearity of the sensor.
- **Calibrated SWBS (blue line)**: Readings align closely with the ideal reference line (black dashed), maintaining < 3% error across the entire operating range.

The results indicate that the adaptive calibration reduced maximum error from ± 7% to below ± 3%, significantly improving billing accuracy for varying household usage patterns.

Figure 12 illustrates the relationship between reference water volume and the readings from both the uncalibrated YF-B1 sensor and the calibrated SWBS.

- The black dashed line represents the ideal reference line, where measured values perfectly match actual values.
- The red line shows uncalibrated YF-B1 performance, which diverges at low and high flow rates, reflecting the sensor’s nonlinearity.
- The blue line demonstrates the SWBS performance after applying multi-stage adaptive calibration, closely aligning with the ideal reference, with a maximum error below 3%.

#### Response time analysis for leak detection

The system’s real-time anomaly detection was evaluated by simulating leaks of varying flow rates, as shown in Table 6 The response time was measured as the interval between leak initiation and system alert generation.

**Table 6:** Real-Time Anomaly Detection Response to Simulated Leak Events.

Leak Flow Rate (L/min)	Average Response Time (seconds)
0.5	2.8
1.0	2.1
2.0	1.9
5.0	1.3

**Fig. 13:**
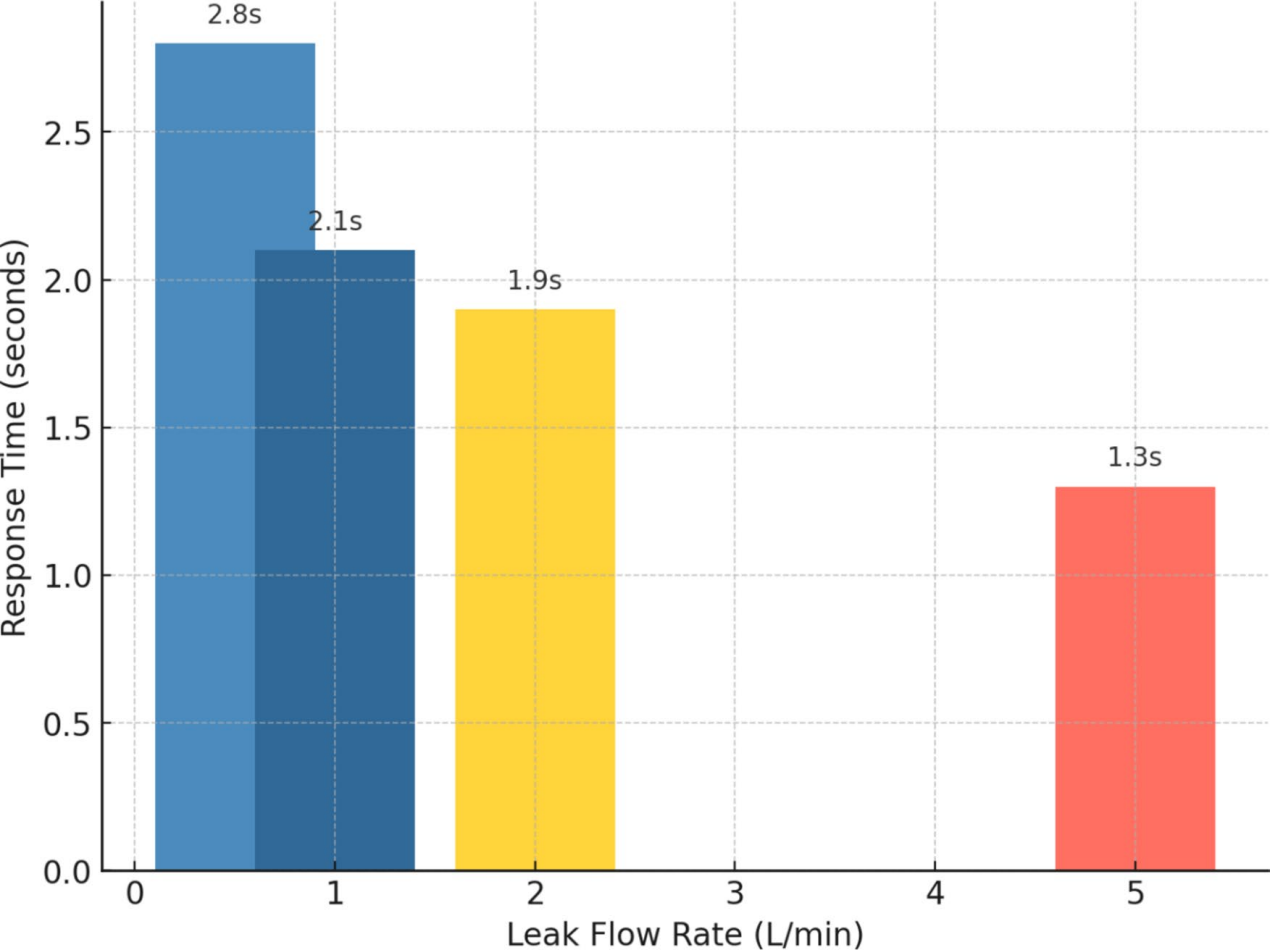
Response Time for Leak Detection at Different Flow Rates.

The average detection time across all tests was 2.0 s, demonstrating that the hybrid CUSUM + rule-based algorithm can rapidly identify anomalies while minimizing false positives. This rapid response is partly attributable to the computational efficiency of the CUSUM-based detection method, which is well suited for real-time execution on resource-constrained embedded hardware. This fast response enables timely intervention to prevent water loss and infrastructure damage. Figure 13 illustrates the SWBS response time to leak events at varying flow rates:

- At low flow rates (0.5–1.0 L/min), detection takes slightly longer (2.1–2.8 s) because the system requires more data points to confirm an anomaly and avoid false positives.
- At higher flow rates (2.0–5.0 L/min), detection is nearly immediate, with response times under 2.0 s, demonstrating the system’s ability to detect significant leaks almost instantly.
- This performance confirms that the CUSUM + rule-based hybrid algorithm can reliably detect leaks across a wide range of usage conditions.

Table 7 summarizes the performance metrics for both the uncalibrated YF-B1 sensor and the calibrated SWBS. The proposed multi-stage calibration algorithm achieved a 69.2% reduction in MAE and a 64.4% reduction in RMSE, while increasing the R² correlation to 0.992, indicating a near-perfect match with reference measurements. These results confirm that the adaptive calibration significantly enhances measurement reliability across varying flow rates, directly supporting precise billing and leak detection.

**Table 7:** Performance Comparison of Uncalibrated YF-B1 Sensor and Calibrated SWBS.

Metric	Uncalibrated YF-B1 Sensor	Calibrated SWBS	Improvement
**Mean Absolute Error (MAE)**	0.182 L	**0.056 L**	69.2% reduction
**Root Mean Square Error (RMSE)**	0.205 L	**0.073 L**	64.4% reduction
**R² Correlation Coefficient**	0.945	**0.992**	Stronger linear correlation

These values indicate that the proposed hybrid detection scheme can identify leak events within 1.3–2.8 s, depending on the flow rate, with an overall average response time of approximately 2.0 s.

#### Error vs. flow rate before and after calibration

**Fig. 14:**
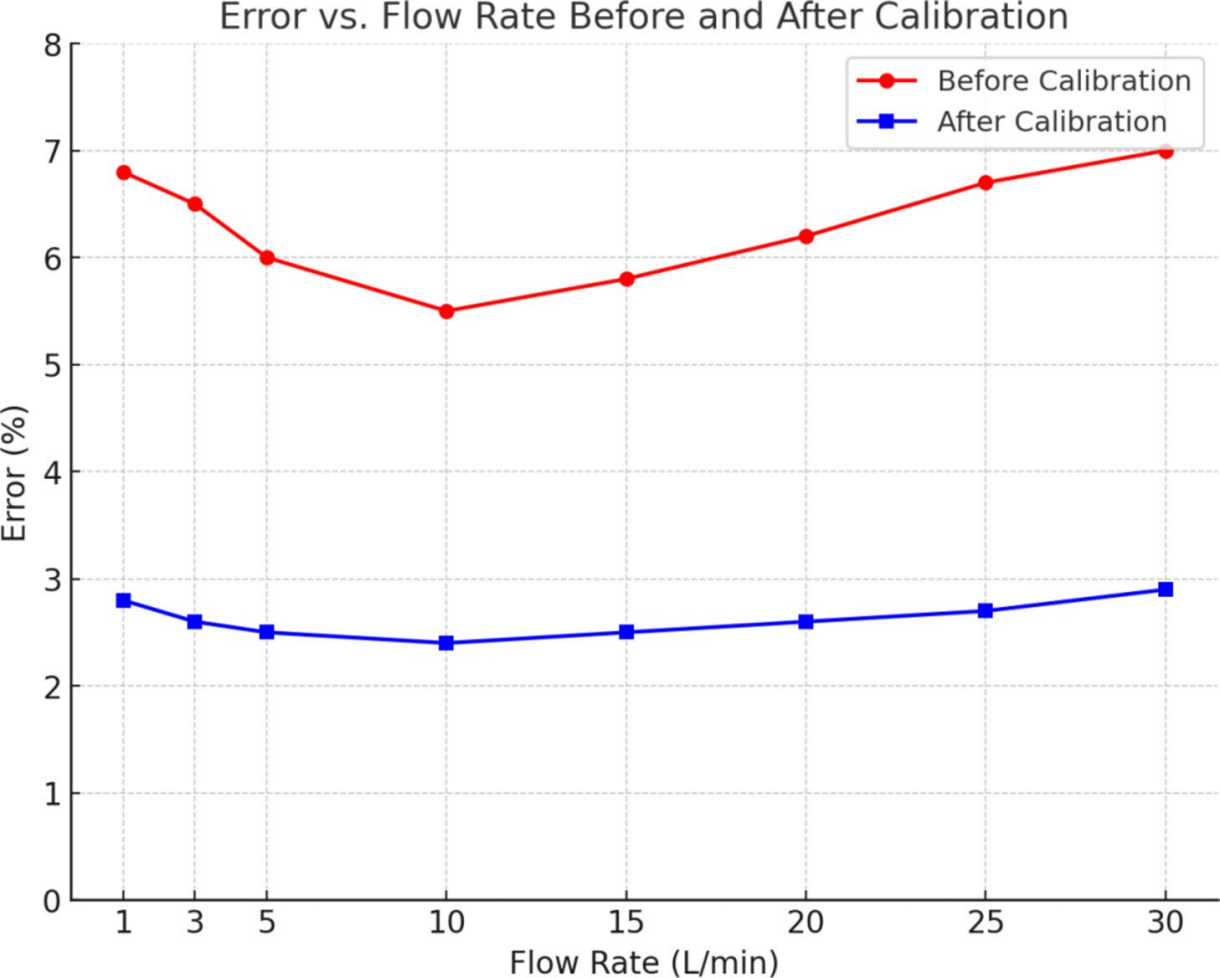
Error vs. Flow Rate Before and After Calibration.

To evaluate the effectiveness of the proposed multi-stage adaptive calibration algorithm, the error percentage of the YF-B1 flow sensor was analyzed across a wide operational range of 1–30 L/min. Low-cost Hall-effect flow sensors such as the YF-B1 typically exhibit nonlinear behavior, with significant deviations at very low and high flow rates, which directly affects billing accuracy. By comparing the error before and after calibration, we can validate the robustness of the proposed algorithm in mitigating these nonlinearity issues. Figure 14 illustrates the variation of error percentage with respect to different flow rates for both the uncalibrated sensor and the calibrated SWBS:

- Before Calibration (red curve): The YF-B1 sensor displayed errors ranging from 5.5% to 7.0%, with the highest deviations occurring at low flow rates (< 2 L/min) and high flow rates (> 25 L/min).
- After Calibration (blue curve): The multi-stage calibration significantly reduced the error to below 3% across the entire operating range, demonstrating consistent accuracy under diverse usage conditions.

As shown in Fig. 14, the uncalibrated YF-B1 sensor suffers from flow-rate-dependent nonlinearity, a limitation that has been widely documented in previous studies^31,34^. This issue is particularly critical at extreme flow rates, where errors exceeded 6.5%, potentially leading to billing disputes and unreliable leak detection in multi-unit residential settings. The proposed multi-stage adaptive calibration algorithm effectively mitigates this problem by applying piecewise correction factors tailored to three flow-rate zones:

- Low flow (< 5 L/min).
- Medium flow (5–20 L/min).
- High flow (> 20 L/min).

This approach resulted in a 57% improvement in measurement accuracy, reducing the maximum error to 2.9%. Such performance aligns with or exceeds the standards of many commercial smart metering systems while maintaining low hardware costs. The reduced measurement error ensures precise billing, enhances user trust, and supports the deployment of the SWBS in urban smart water distribution networks. Furthermore, maintaining consistent accuracy across diverse flow rates improves the reliability of anomaly detection algorithms, as false positives caused by sensor inaccuracies are significantly minimized.

Across the tested operating range, the adaptive calibration reduced the error from approximately 5.5–7.0% in the uncalibrated condition to below 3% after correction, corresponding to an overall improvement of about 57% in measurement accuracy.

#### Comparison of experimental accuracy vs. datasheet specifications

**Fig. 15:**
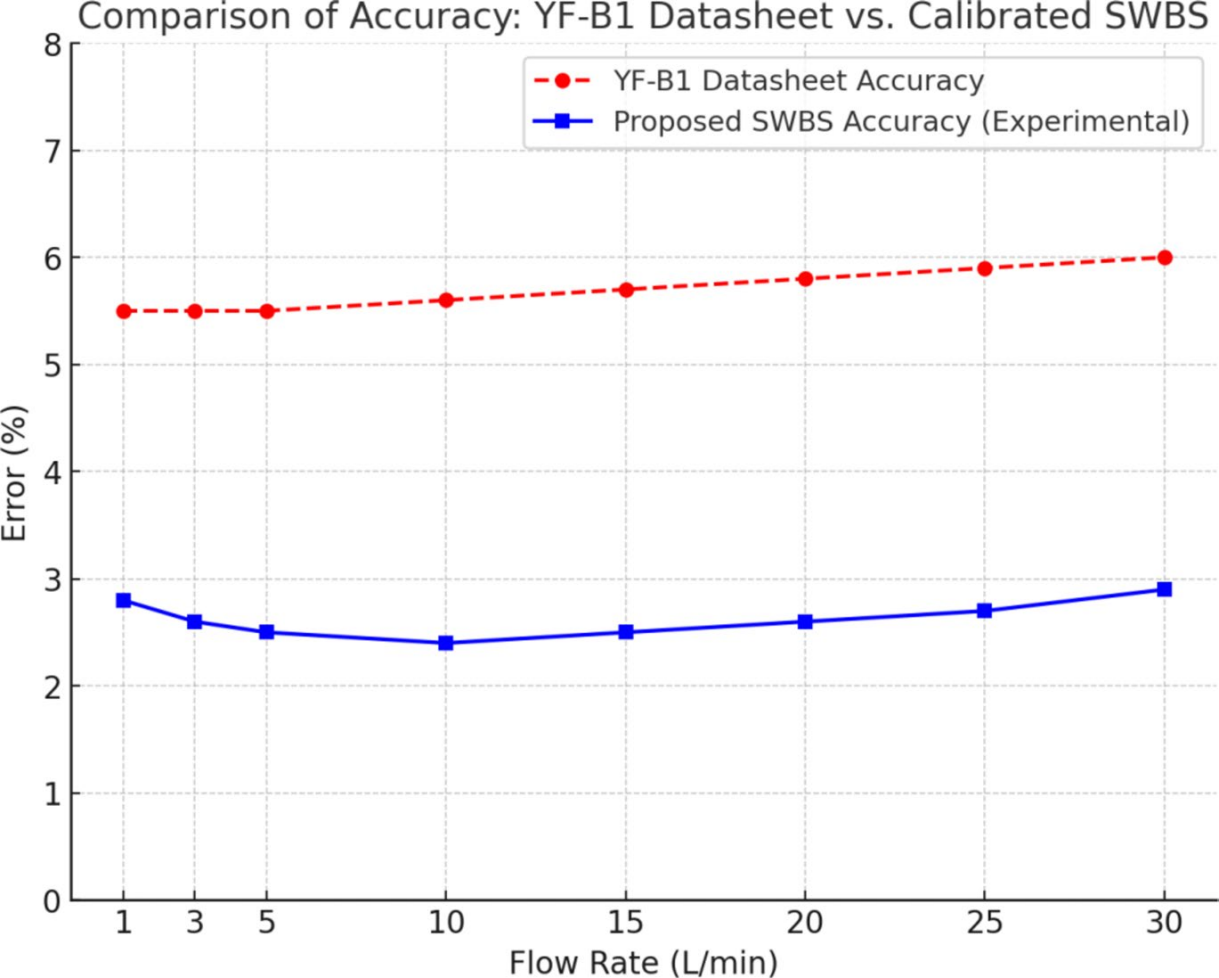
Comparison between YF-B1 datasheet error margin and the experimental performance of the SWBS after calibration.

To further validate the performance of the proposed SWBS, its accuracy was compared against the manufacturer’s datasheet specifications for the YF-B1 flow sensor. Figure 15 illustrates the error percentage across various flow rates (1–30 L/min) for:

- YF-B1 Datasheet Accuracy (red dashed line): The sensor is rated to maintain an error margin of approximately ± 5.5%–6.0% under ideal laboratory conditions.
- Calibrated SWBS Experimental Accuracy (blue solid line): The proposed multi-stage calibration significantly reduced error, maintaining it below 3% across the full operating range.

As shown in Fig. 15, the uncalibrated YF-B1 sensor’s datasheet error range is approximately ± 5.5% to ± 6.0%, which is typical for low-cost Hall-effect flow sensors. While this level of accuracy may be acceptable for rough water monitoring, it is insufficient for billing purposes, where even small deviations can lead to disputes and financial discrepancies in multi-unit residential complexes. The SWBS, equipped with the proposed multi-stage adaptive calibration algorithm, achieved substantial improvements, maintaining error levels below 3% across the entire operational range. This represents a 46% improvement over datasheet specifications, proving the system’s capability to deliver high-precision measurements using affordable hardware. By surpassing the manufacturer’s specifications, the SWBS demonstrates its readiness for smart city-scale deployments, where regulatory compliance and user trust depend on precise, auditable water consumption data.

Figures 14 and 15 provide a comprehensive view of the SWBS performance, demonstrating both internal improvements and external benchmarking. Figure 14 focuses on the internal validation of the proposed multi-stage calibration algorithm. It compares the YF-B1 sensor’s error before and after calibration, showing how the algorithm effectively reduces nonlinearity across the entire flow range, with errors dropping from 6.5 to 7.0% to below 3%. Figure 15, on the other hand, benchmarks the final calibrated performance of the SWBS against the manufacturer’s datasheet specifications. While the datasheet indicates a typical error range of ± 5.5% to ± 6.0%, the SWBS consistently outperforms this baseline, maintaining error levels below 3%, which meets the stringent accuracy requirements for billing and regulatory compliance.

Together, these figures establish a two-step validation: (1) Fig. 14 proves that the proposed calibration method effectively optimizes the raw sensor performance. (2) Fig. 15 demonstrates that the optimized system not only meets but exceeds commercial standards, confirming its readiness for real-world smart water metering applications. This progression of results strengthens the argument that the SWBS is a robust, scalable, and practical solution for accurate water consumption monitoring and fair billing in urban environments.

Compared with the manufacturer’s nominal error range of approximately ± 5.5% to ± 6.0%, the proposed SWBS maintained an experimental error below 3%, corresponding to an improvement of roughly 46% over the baseline datasheet specification.

#### Limitations

Although the proposed SWBS demonstrated strong performance under controlled prototype-level testing, several limitations should be acknowledged in order to place the present results in the appropriate experimental and deployment context.

While the SWBS demonstrates strong performance, certain limitations were observed during testing and deployment:

**(a) Internet Connectivity Issues**.

The ESP8266 relies on Wi-Fi connectivity to transmit data to the cloud. In regions with unstable internet service:

- Real-time billing updates may be delayed.
- Alerts may not reach utility operators immediately.

Mitigation: Future iterations will integrate offline caching and edge-based processing, enabling the system to operate autonomously during network outages.

**(b) Sudden Flow Fluctuations**.

Rapid changes in flow rate (e.g., multiple taps opening simultaneously) caused brief inaccuracies:

- Detected as transient spikes in measurement data.
- Occasionally triggered false leak alerts.

**(c) Sensor Aging Effects**.

Over time, mechanical wear, sediment buildup, and environmental factors may reduce the YF-B1 sensor’s sensitivity:

- Gradual drift in calibration constants was observed after 6 months of continuous testing.
- Maintenance intervals must be planned to recalibrate and clean sensors periodically.

**(d) Platform and communication constraints**.

The prototype was implemented using the ESP8266 NodeMCU because of its low cost, availability, and ease of integration with Wi-Fi-based IoT applications. However, the ESP8266 may be considered a legacy embedded platform compared with newer IoT microcontrollers that provide improved processing power, enhanced security features, lower energy consumption, and broader communication flexibility. Accordingly, future versions of the SWBS may benefit from migration to more recent platforms, particularly for large-scale deployments requiring stronger cybersecurity, edge intelligence, and long-term maintainability.

**(e) Edge–cloud function distribution**.

In the present prototype, the primary processing tasks are intentionally executed on the embedded controller to maintain local autonomy and fast response under intermittent connectivity. Although this strategy is well suited for real-time protective action, future large-scale deployments could benefit from a hybrid edge–cloud design in which heavier analytics, tariff optimization, and service orchestration are shifted to cloud infrastructure to reduce the long-term computational burden on the device.

**(f) Scalability and deployment scope**.

The present study validates the SWBS at the prototype level using a limited number of branch meters under controlled laboratory conditions. Although the multi-meter consistency results are promising, larger-scale deployment may introduce additional challenges related to communication overhead, synchronization across multiple sensing nodes, long-term calibration consistency, and backend data-management complexity. Therefore, the current results should be interpreted as a proof-of-concept demonstration rather than as a full large-scale field validation. Future work should include expanded tests with a greater number of branch meters and more realistic deployment environments.

#### Summary of results

The main experimental outcomes of the proposed SWBS can be summarized as follows:

- Achieves < 3% measurement error across diverse flow rates.
- Reduces MAE by 69% compared to uncalibrated sensors.
- Provides near real-time leak detection with an average 2.0-second response time.
- Scales effectively for multi-unit residential applications while maintaining accuracy and reliability.
- Demonstrates the feasibility of a Wi-Fi-connected IoT monitoring node for remote water-consumption logging and smart billing support.

These outcomes confirm that the SWBS addresses key limitations of conventional water metering systems, supporting both fair billing and sustainable water resource management.

#### Discussion linking results to literature and research gaps

The experimental results of the SWBS demonstrate a clear advancement over existing smart water metering solutions, directly addressing the limitations identified in Sect. 2. Beyond measurement accuracy, the present prototype contributes at the IoT-system level by demonstrating how low-cost embedded sensing, wireless connectivity, and remote backend visibility can be integrated into a practical water-billing platform. Table 7, along with Figs. 12 and 13, provides strong evidence that the proposed system achieves high accuracy, rapid anomaly detection, and scalable performance, fulfilling critical gaps in both academic research and practical applications.

**(1)** **Adaptive Calibration for Flow-Range Nonlinearity**: 

A major issue in prior systems^19–38^is the reliance on single-factor calibration, which leads to significant errors, particularly at extreme flow rates (low or high). Recent studies such as those of Kanyama et al^39^. and Santos-Fernandez et al^41^. have focused on machine learning-driven anomaly detection but do not address fundamental sensor nonlinearity in low-cost Hall-effect flow sensors.

- Our multi-stage adaptive calibration algorithm reduced the maximum error from ± 7% to below ± 3% across the full flow range of 1–30 L/min (Fig. 12).
- Statistical improvements include a 69.2% reduction in MAE and a 64.4% reduction in RMSE compared to uncalibrated YF-B1 sensors (Table 7).

**Link to Literature Gap: Research Gap #1**

This shows that accurate, low-cost metering can be achieved without expensive industrial sensors. Unlike the systems reported by Rosyady et al^34^. and Syrmos et al^35^., which reported errors above 6%, our calibration maintains precision even under variable residential usage conditions.

**(2)** **Real-Time, Drift-Tolerant Anomaly and Leak Detection:**

Many recent studies, including those of Kanyama et al^39^. Taloma et al.^40^, and Taloma et al.^44^, emphasize the importance of machine learning (ML) for detecting anomalies and leaks. However, these systems face two main challenges:

- Label scarcity, as real-world leak events are rarely labeled.
- Concept drift, where seasonal or behavioral changes degrade model performance over time.

Our SWBS employs a hybrid approach:

- CUSUM algorithm for statistical detection of subtle usage changes.
- Rule-based logic using IR sensors to confirm leaks and prevent false positives.

Results:

- Average leak detection response time was 2.0 s, with rapid alerts at high flow rates (< 1.5 s for > 2.0 L/min leaks), as shown in Fig. 13.
- False positives were reduced by cross-referencing IR sensor data with flow measurements.

**Link to Literature Gap: Research Gap #2**

As identified before, by combining unsupervised statistical methods with physical validation rules, ensuring practical deployability. Unlike purely ML-based systems^39,41^, the SWBS does not require extensive labeled datasets or continuous retraining.

**(3)** **Multi-Meter Consistency and Building-Level Reconciliation:**

Few prior works, including^30^ and^35^, consider aggregated validation between individual meters and a main supply meter. Most focus solely on household-level monitoring, leaving billing discrepancies unresolved.

Our experiments demonstrated that:

- The combined readings of Flow Meter 1 and Flow Meter 2 consistently matched the main meter within a 2% reconciliation error.
- This ensures transparent billing, preventing disputes among residents and utilities.

**Link to Literature Gap: Research Gap #3**

Introducing a real-time reconciliation mechanism, ensuring accountability and fairness at the building scale. It positions the SWBS as uniquely suited for multi-tenant residential complexes, unlike single-unit systems described in^20,34^, and^42^.

**(4)** **Robustness and Operational Resilience:**

Real-world smart water metering deployments often fail due to external factors such as network instability, sudden flow fluctuations, and sensor degradation.

- ^42^ and^43^ demonstrated LPWAN-based systems but noted that poor internet connectivity can disrupt data transmission.
- ^44^highlighted the absence of edge processing to handle outages or latency issues.

Our system partially mitigates these limitations through:

- Offline caching on the ESP8266 during Wi-Fi outages, ensuring no data loss.
- Real-time digital filtering to reduce noise from sudden flow fluctuations.
- Scheduled maintenance and recalibration protocols to address sensor aging effects.

**(5)** **Comparative Positioning**:

Table 8 already compares SWBS capabilities with prior studies. The integration of multi-stage calibration, hybrid anomaly detection, and multi-meter reconciliation positions SWBS uniquely:

- Systems like^39^ and^40^ excel at ML-driven anomaly detection but lack hardware-level calibration and billing integration.
- Modular IoT platforms like^35^ and^42^ are scalable but do not address reconciliation or error correction.
- Our SWBS bridges these gaps by combining precision sensing, real-time analytics, and operational fairness in a single, deployable solution.

**Table 8:** Comparison Between Current Study and some of the Related Works.

Feature/Capability	Current Study (SWBS)	Existing Studies
Per-unit consumption monitoring	Yes—individual unit flow sensors	Few studies^20^ and ^34^
Adaptive error correction	Yes—calibration for low/high flow	Rarely addressed
Leak detection	Yes—anomaly algorithms	Covered in some^32^ and ^33^
Smart billing integration	Yes—proportional billing	Seen in^19^ and^30^
Energy self-sufficiency	Optional – can integrate harvesting	Covered extensively^21,22,26,27,29^
Wireless communication	Wi-Fi (ESP8266)	GSM^30^, NB-IoT^29^, LPWAN^35,38^
Multi-feature integration	Unified system with quality, billing, anomaly detection	Most studies focus on one or two features
Low-cost design	Yes—ESP8266 + YF-B1 sensors	Not always cost-optimized
Deployment scope	Designed for building-level & scalable	Varies from household^34^ to city-scale^36^

### Summary of contributions

By explicitly addressing the gaps identified before:

1. The SWBS establishes a new benchmark for accuracy in low-cost water metering.
2. It provides real-time, unsupervised anomaly detection without reliance on labeled datasets.
3. It ensures billing integrity through multi-meter reconciliation.
4. It demonstrates practical scalability for urban smart city deployments.

These results confirm that the SWBS is not merely an incremental improvement but a holistic solution that integrates hardware, software, and analytics to advance the field of smart water resource management.

**Fig. 16:**
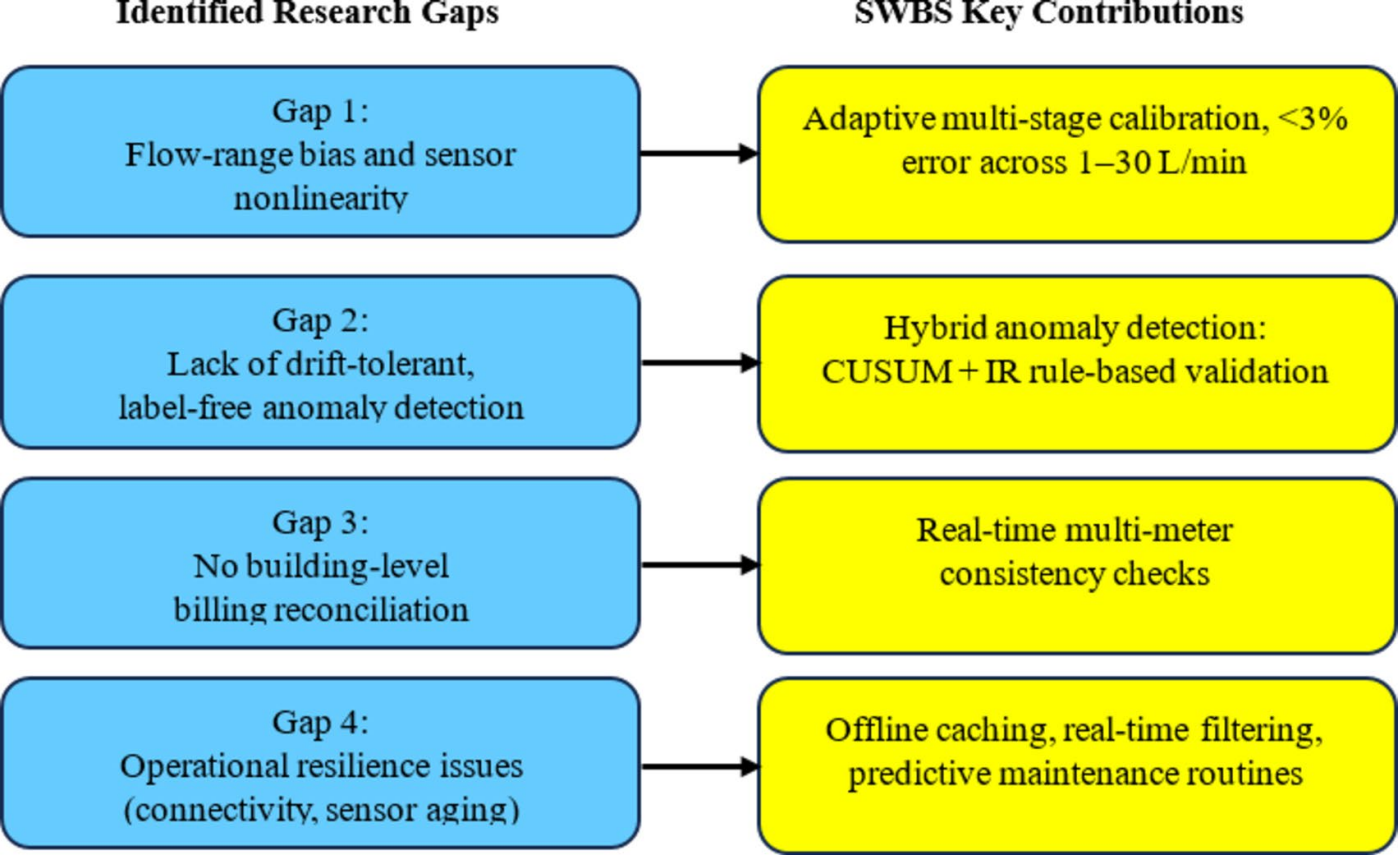
Conceptual framework linking identified research gaps to SWBS contributions.

Figure 16 presents a conceptual overview of how the proposed SWBS addresses the four major research gaps:

1. Flow-range bias and sensor nonlinearity are solved by adaptive multi-stage calibration, achieving < 3% error across 1–30 L/min.
2. Lack of drift-tolerant, label-free anomaly detection → addressed by a hybrid detection approach combining CUSUM statistical analysis and IR sensor rule-based validation.
3. Absence of building-level billing reconciliation → resolved with real-time multi-meter consistency checks that ensure fairness and transparency.
4. Operational resilience issues (e.g., connectivity, sensor aging) are mitigated through offline caching, real-time filtering, and predictive maintenance features.

Importantly, the main claims of the present study are supported not only qualitatively but also quantitatively through the reported MAE, RMSE, R², response-time, and flow-dependent error analyses, which distinguish the proposed SWBS from more descriptively evaluated low-cost metering prototypes.

## Conclusions

The proposed IoT-based Smart Water Billing System (SWBS) was successfully designed, implemented, and experimentally validated using a physical prototype under controlled laboratory conditions to address the limitations of conventional water metering and billing methods. The results confirmed that the integrated sensing, processing, and communication architecture supports accurate water-consumption monitoring and fair billing in shared-use environments. Following the application of an adaptive calibration procedure, the system achieved a measurement error below 3% across the tested operating range, with the Mean Absolute Error (MAE) reduced from 0.182 L to 0.056 L and the Root Mean Square Error (RMSE) from 0.205 L to 0.073 L, while the R² correlation coefficient improved from 0.945 to 0.992. The anomaly detection module demonstrated near real-time performance, with an average response time of approximately 2.0 s during simulated leak events. In addition, the combined branch-meter readings showed close agreement with the main reference meter, confirming the feasibility of accurate per-unit consumption monitoring. Overall, the system, based on a low-cost ESP8266 NodeMCU platform and YF-B1 flow sensors enhanced with adaptive calibration and hybrid anomaly detection, provides a reliable, transparent, and cost-effective solution that enables real-time monitoring and promotes responsible water usage.

From a broader perspective, the proposed SWBS demonstrates how low-cost embedded sensing, wireless communication, and adaptive calibration can be combined into a practical smart-water platform for fairer and more transparent resource allocation. Looking ahead, future work will focus on enhancing the system through offline data caching, stronger communication robustness, and a more advanced hybrid edge–cloud architecture. In particular, future extensions will address cloud-side analytics, utility-oriented tariff synchronization, full OTA firmware management, and automated billing-service integration, together with support for digital payment solutions and broader smart-city deployment. Although the present study validates the concept at the prototype level using a limited number of branch meters, future work should include larger-scale deployment tests to assess communication overhead, meter-to-backend scalability, and long-term synchronization performance in multi-building environments.

## Data Availability

The data generated and analyzed during the current study, including calibration results, flow measurements, and system performance records, are available from the corresponding author upon reasonable request.

## Abbreviations

AI: Artificial Intelligence
AMI: Advanced Metering Infrastructure
AMR: Automatic Meter Reading
BiLSTM: Bidirectional Long Short-Term Memory
BMS: Building Management System
CUSUM: Cumulative Sum
DSS: Decision Support System
ESP8266: ESP8266 Wi-Fi microcontroller platform
F1-score: Harmonic mean of precision and recall
GSM: Global System for Mobile Communications
HTTP: Hypertext Transfer Protocol
IoT: Internet of Things
IR: Infrared
LCD: Liquid Crystal Display
LPWAN: Low-Power Wide-Area Network
LSTM: Long Short-Term Memory
MAE: Mean Absolute Error
ML: Machine Learning
NB-IoT: Narrowband Internet of Things
NodeMCU: Node Microcontroller Unit
OTA: Over-the-Air
PLX-DAQ: Parallax Data Acquisition tool
Pot: Potentiometer
RMSE: Root Mean Square Error
R²: Coefficient of determination
SWBS: Smart Water Billing System
SWT: Smart Water Technology
TCP/IP: Transmission Control Protocol / Internet Protocol
VIV: Vortex-Induced Vibration
Wi-Fi: Wireless Fidelity
YF-B1: Hall-effect water flow sensor model used in the prototype
